# Effects of Photoperiod and Light Quality on Phlorotannin Accumulation and Multi-Omics Responses in *Sargassum muticum*

**DOI:** 10.3390/md24090330

**Published:** 2026-09-21

**Authors:** Fang Lü, Yan Li, Dongmei Zhan, Haiyi Wu

**Affiliations:** 1 Shandong Engineering Research Center of Nearshore Seaweed Bed Building, Marine Science Research Institute of Shandong Province (National Oceanographic Center, Qingdao), Qingdao 266104, China; 2 School of Fisheries, Ludong University, Yantai 264025, China

**Keywords:** *Sargassum muticum*, phlorotannins, photoperiod, light quality, transcriptomics, metabolomics

## Abstract

Brown algae contain phlorotannins with recognized biological and ecological functions, but the responses of phenolic metabolism to different light environments remain incompletely understood. In this study, *Sargassum muticum* was cultivated for 15 days under different photoperiods (12L:12D, 16L:8D, and 20L:4D) and light qualities (white, blue, and red light). Growth, photosynthetic performance, oxidative stress indicators, and phlorotannin content were examined, together with transcriptomic and untargeted metabolomic responses. Extended photoperiods were associated with increased growth and a transient increase in phlorotannin content, whereas blue light reduced growth but maintained relatively high chlorophyll *a* content and photosynthetic efficiency. Transcriptomic analysis identified 10,322, 3790, and 9301 differentially expressed genes under long-photoperiod, blue-light, and red-light treatments, respectively, including 388 genes shared among the three comparisons. Seven transcripts were putatively annotated as polyketide synthases, including four candidate type III polyketide synthase transcripts, and 26 transcripts were annotated as candidate acetyl-CoA carboxylase-related sequences. Untargeted LC–MS analysis yielded 949 putatively annotated metabolites, among which features annotated as salicylic acid and γ-glutamylcysteine showed increased relative abundance under the three light treatments. A feature putatively annotated as phloroglucinol did not differ significantly among treatments. Correlation analysis indicated coordinated, but non-causal, associations between light-responsive transcripts and changes in amino acid, carbohydrate, and lipid metabolism. These results characterize the physiological and multi-omics responses of *S. muticum* to altered light regimes and provide candidate molecular targets for future functional and targeted chemical validation.

## 1. Introduction

*Sargassum muticum*, a large brown alga widely distributed along the northwest Pacific coast, is a dominant species in nearshore seaweed beds [1]. In marine ecosystems, *S. muticum* sustains coastal biodiversity and provides ecosystem services such as water purification and carbon sequestration [2,3]. Notably, *S. muticum* is rich in diverse bioactive substances (including alginate, fucoxanthin, and phlorotannins), among which phlorotannins are particularly abundant and biologically active [4], highlighting its potential as a source of marine-derived bioactive compounds [5,6].

Phlorotannins are a class of secondary metabolites that are found exclusively in marine brown algae. These compounds consist of phloroglucinol units, which are connected through ether bonds, carbon–carbon bonds, or a combination of both [7,8]. The molecular weight of phlorotannins varies widely, from about 0.126 to 650 kDa. This structural diversity gives rise to a broad range of biological activities, including antioxidant, antibacterial, anti-inflammatory, antitumor, and antidiabetic effects [9,10,11]. In brown algae, phlorotannins are known to fulfill several physiological and ecological roles. They function as effective antioxidants by scavenging reactive oxygen species through their phenolic hydroxyl groups, which protects algal tissues from oxidative damage [12,13]. Additionally, phlorotannins contribute to the reinforcement of cell walls through crosslinking with polysaccharides [14]. Due to their strong absorbance of UV radiation, these compounds also serve as natural UV screens [15]. Phlorotannins participate in chemical defense as well, since they can deter fouling organisms and grazers [16,17].

The biosynthetic pathway of phlorotannins is markedly different from that of phenolic compounds in higher plants. Higher plants synthesize phenolics via the shikimate and phenylpropanoid pathways. Brown algae, by contrast, utilize a polyketide pathway for phlorotannin production [18]. Current evidence indicates that the phloroglucinol monomer is synthesized through the acetate–malonate pathway, involving acetyl-CoA carboxylase and type III polyketide synthase [18,19]. Nevertheless, the polymerization steps that convert monomers into high-molecular-weight phlorotannins remain poorly understood [20]. The precise enzyme systems responsible for this process, along with the regulatory networks involved, have not yet been elucidated.

Environmental factors exert a strong influence on the accumulation of phlorotannins. Among these factors, light conditions appear to be particularly critical. Phlorotannin content shows pronounced seasonal variation in natural populations, and this variation correlates with sunlight intensity [21,22,23]. Experimental studies have demonstrated that increasing light intensity can promote phlorotannin accumulation [24]. Light quality also has specific effects on phlorotannin production, with blue light and ultraviolet radiation having been identified as effective inducers [25]. In the intertidal habitat, *S. muticum* is exposed to dramatic fluctuations in light between submersion and aerial exposure, which are thought to be associated with dynamic patterns of phlorotannin accumulation [26].

Although light-responsive accumulation of phlorotannins has been reported, the underlying molecular mechanisms remain largely unclear. In higher plants, light signals are perceived by photoreceptors and subsequently activate transcription factors that regulate genes involved in secondary metabolism [27]. Brown algae possess diverse photoreceptors, including canonical plant types and novel receptors such as aureochromes [28,29,30]. How light signals are transmitted to genes involved in phlorotannin biosynthesis, however, has not been clarified. In *Saccharina japonica*, Li et al. [24] reported that a type III PKS gene showed light-intensity-dependent expression accompanied by elevated phlorotannin content, and several candidate transcription factors were predicted. Nevertheless, the complexity of algal photoreceptor networks and potential cross-talk among pathways [28] suggest that multilayered regulatory mechanisms are likely involved. To address these knowledge gaps, integrative multi-omics approaches are required. Combining transcriptomics with metabolomics allows for comprehensive profiling of gene expression dynamics and metabolite changes, which can help identify candidate genes and metabolic pathways associated with light-responsive phlorotannin accumulation.

In the present study, thalli of *S. muticum* were cultured for 15 days under different photoperiods (12L:12D, 16L:8D, and 20L:4D) and light qualities (white, red, and blue). Growth, photosynthetic performance, oxidative stress indicators, and phlorotannin content were examined. Representative treatments were further analyzed using transcriptomic and untargeted metabolomic methods to characterize light-responsive changes in gene expression and metabolic profiles. Particular attention was given to transcripts putatively annotated as PKS and ACC because of their possible relationships with malonyl-CoA and polyketide metabolism. The study was designed to identify physiological and molecular associations with phlorotannin biosynthesis under different light conditions in brown algae.

## 2. Results

### 2.1. Physiological Responses

#### 2.1.1. Growth Performance

**Figure 1:**
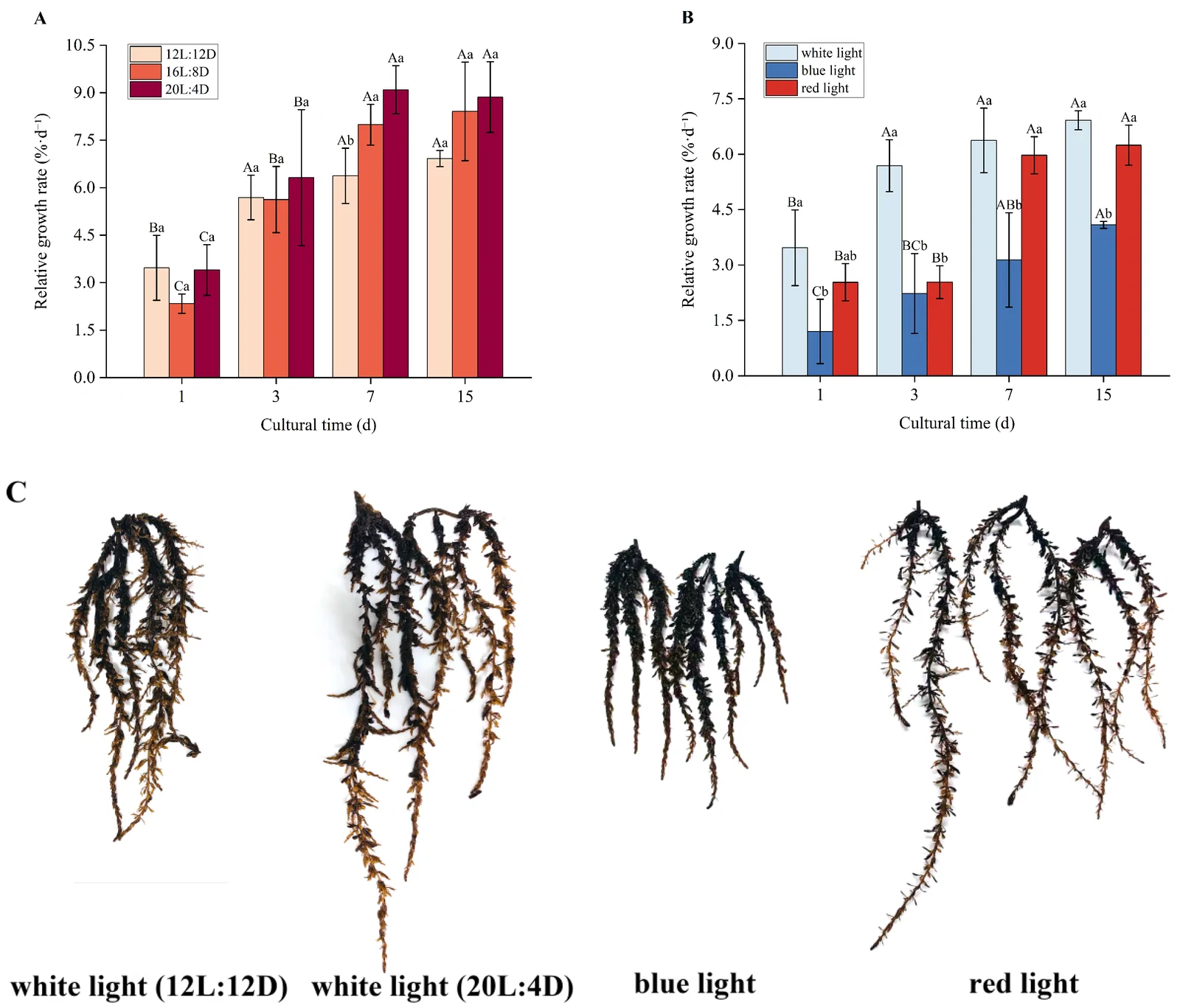
Effects of photoperiod and light quality on the relative growth rate and morphology of *Sargassum muticum*: (**A**) Relative growth rate under different photoperiods. (**B**) Relative growth rate under different light qualities. Data are presented as mean ± standard deviation (*n* = 3). Different uppercase letters indicate significant differences among time points within the same treatment, and different lowercase letters indicate significant differences among treatments at the same time point (*p* < 0.05; one-way ANOVA followed by Tukey’s HSD test). (**C**) Morphological characteristics of *S. muticum* after 7 days of culture under different light conditions.

Over the 15-day cultivation, the relative growth rate (RGR) of *S. muticum* exhibited distinct dynamics across different photoperiods (Figure 1A). All treatments increased during the initial phase, but the 12L:12D group plateaued by day 3, whereas the 16L:8D and 20L:4D groups continued rising until day 7. Significant intergroup differences emerged only on day 7, when the 12L:12D group was significantly lower than both the 16L:8D and 20L:4D groups (*p* < 0.05), with no difference between the latter two treatments.

RGR was also significantly affected by light quality (Figure 1B). Under white light, RGR peaked by day 3 and stabilized thereafter; red light showed a similar trajectory but reached a maximum by day 7. Blue light led to a continuous but slower increase throughout the experiment. From day 7 onward, the blue-light group exhibited significantly lower RGR than both the white- and red-light groups (*p* < 0.05), while no differences were detected between white and red light.

Visual assessments revealed clear morphological divergence among light quality treatments by day 7 (Figure 1C). White light thalli displayed uniformly sized blades and moderate internodal spacing. Blue light thalli appeared more compact, with smaller, more densely arranged blades and darker pigmentation. Red light induced elongated branches with thinner blades, wider spacing, and paler coloration.

#### 2.1.2. Photosynthetic Pigments

**Figure 2:**
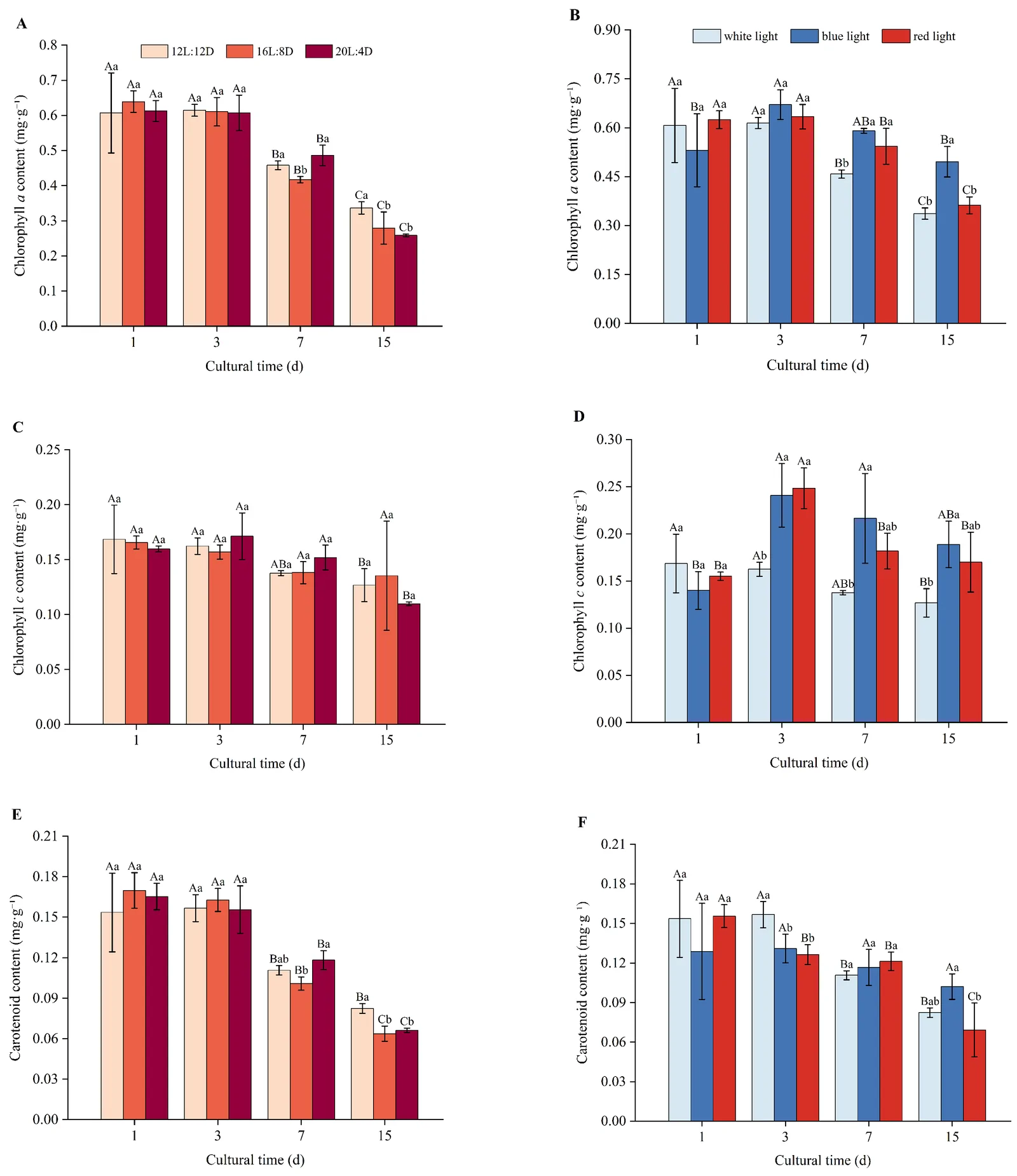
Effects of photoperiod and light quality on photosynthetic pigment content of *Sargassum muticum* during 15 days of cultivation. Photoperiod effects on chlorophyll *a* (**A**), chlorophyll *c* (**C**), and carotenoid (**E**) content. Light quality effects on chlorophyll *a* (**B**), chlorophyll *c* (**D**), and carotenoid (**F**) content. Different uppercase letters indicate significant differences among time points within the same treatment, and different lowercase letters indicate significant differences among treatments at the same time point (*p* < 0.05; one-way ANOVA followed by Tukey’s HSD test).

Under different photoperiods, all three pigment types exhibited broadly similar temporal patterns: relatively stable through days 1–3, followed by progressive declines from day 7 onward (Figure 2A,C,E). By day 15, chlorophyll *a* had decreased by approximately 55–65% relative to initial values across all groups, with the 12L:12D group retaining significantly higher levels than the 16L:8D and 20L:4D groups (*p* < 0.05). Carotenoids followed a similar trajectory, and the 12L:12D group again exhibited the highest retention at day 15 (*p* < 0.05). Chlorophyll *c* declined more modestly, decreasing significantly only by day 15 in the 12L:12D and 20L:4D groups, with no significant intergroup differences at any time point.

Light quality exerted more differentiated effects on pigment dynamics (Figure 2B,D,F). Most notably, blue light sustained pigment levels more effectively than white or red light across all three pigment types. Chlorophyll *a* under blue light increased transiently on day 3 (*p* < 0.05) before gradually declining, whereas the white- and red-light groups declined steadily from day 7. By day 15, the blue-light group retained approximately 1.5–1.7-fold higher chlorophyll *a* than the white- and red-light groups (*p* < 0.05). Similarly, chlorophyll *c* peaked under blue and red light on day 3 while declining continuously under white light; the blue-light group maintained significantly higher values on days 3, 7, and 15 (*p* < 0.05). Carotenoids remained stable throughout under blue light, contrasting with progressive declines under white (from day 7) and red light (from day 3); by day 15, the blue-light group was significantly higher than the red-light group (*p* < 0.05). Detailed pairwise comparisons at each time point are indicated by letters in Figure 2.

#### 2.1.3. Chlorophyll Fluorescence

**Figure 3:**
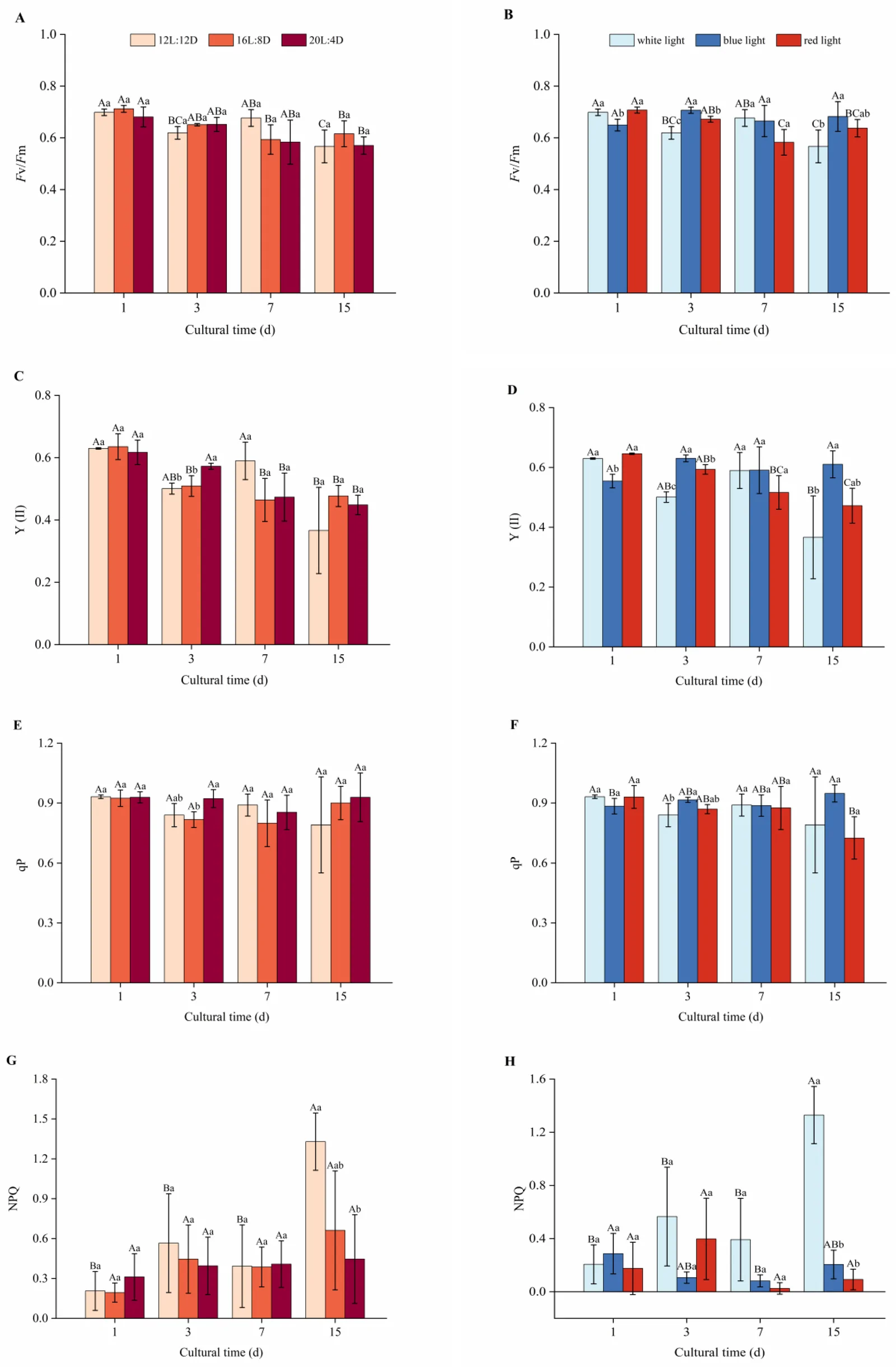
Effects of photoperiod and light quality on chlorophyll fluorescence parameters of *Sargassum muticum* during 15 days of cultivation. Photoperiod effects on (*F*v/*F*m) (**A**), Y(II) (**C**), qP (**E**), and NPQ (**G**). Light quality effects on (*F*v/*F*m) (**B**), Y(II) (**D**), qP (**F**), and NPQ (**H**). Different uppercase letters indicate significant differences among time points within the same treatment, and different lowercase letters indicate significant differences among treatments at the same time point (*p* < 0.05; one-way ANOVA followed by Tukey’s HSD test).

Under different photoperiods, the maximum quantum yield of PSII (*F*v/*F*m) showed a general declining trend, falling from initial values of approximately 0.72–0.75 to 0.55–0.65 by day 15 (Figure 3A). The 12L:12D group exhibited fluctuations with declines at days 3 and 15, whereas 16L:8D and 20L:4D declined more continuously from day 7; no significant intergroup differences were detected. Among the other parameters, the effective quantum yield [Y(II)] under 20L:4D was significantly higher than the other groups on day 3 (*p* < 0.05; Figure 3C); photochemical quenching (qP) remained relatively stable across all photoperiods (Figure 3E); and non-photochemical quenching (NPQ) diverged at day 15, when the 12L:12D group increased significantly above the 20L:4D group (*p* < 0.05; Figure 3G), indicating enhanced thermal dissipation under the standard photoperiod.

Light quality produced more pronounced differentiation in fluorescence parameters (Figure 3B,D,F,H). *F*v/*F*m under blue light remained stable near initial levels (~0.70–0.75) throughout the cultivation, whereas the white- and red-light groups declined to ~0.55–0.60 by day 15. By day 3, a clear ranking had emerged (blue > red > white; *p* < 0.05), and at day 15, blue light still maintained significantly higher *F*v/*F*m than white light (*p* < 0.05). Y(II) patterns paralleled *F*v/*F*m, with blue light sustaining effective photochemical efficiency while the red- and white-light groups declined over time. qP increased under blue light by day 15 but decreased under red light; white light remained stable. NPQ increased markedly only under white light at day 15, reaching values significantly higher than those under both blue and red light (*p* < 0.05), consistent with greater reliance on thermal energy dissipation. Together, these results indicate that blue light maintained superior PSII photochemical performance throughout the experiment.

#### 2.1.4. Oxidative Stress Markers

**Figure 4:**
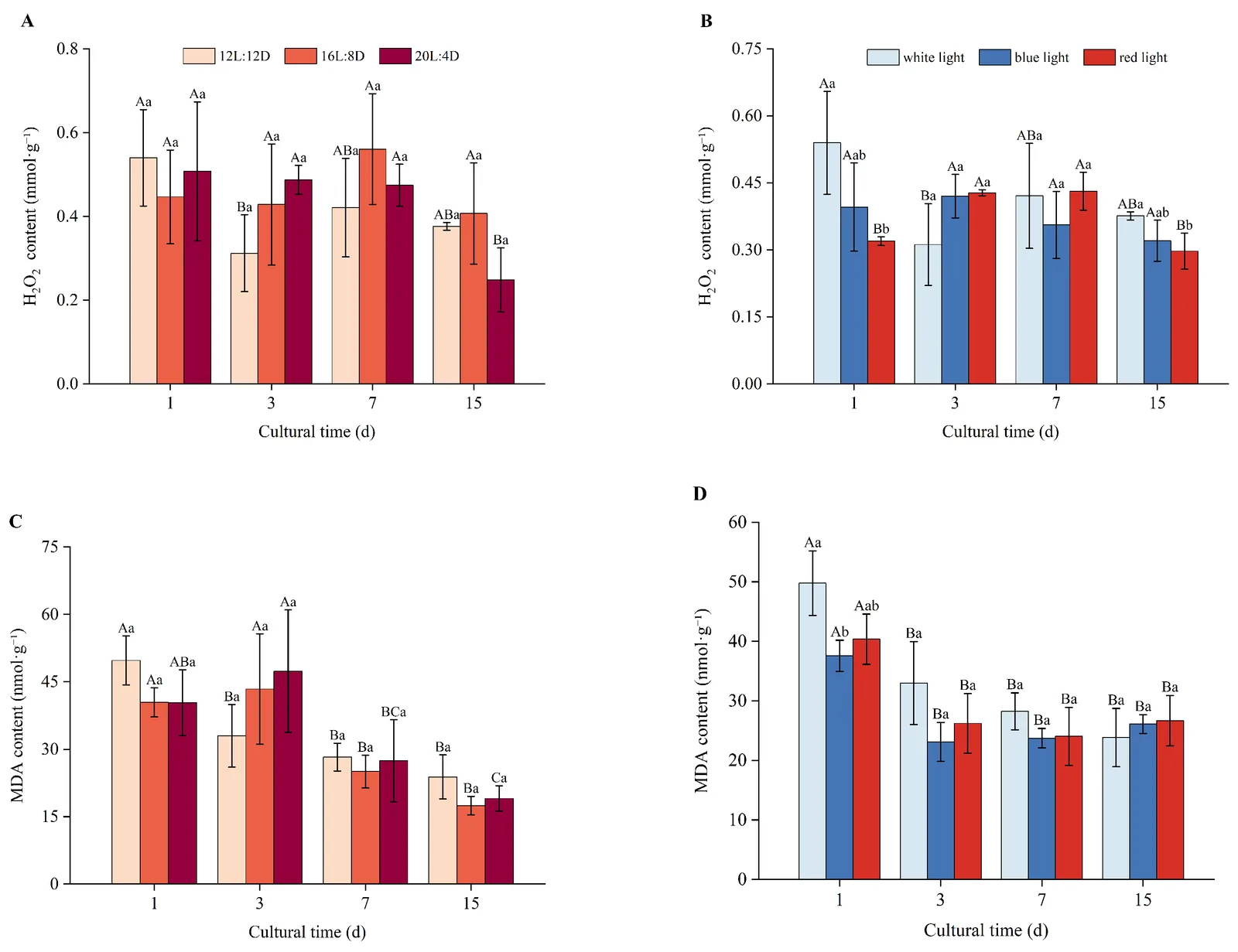
Effects of photoperiod and light quality on oxidative stress indicators of *Sargassum muticum* during 15 days of cultivation. Photoperiod effects on H_2_O_2_ content (**A**) and MDA content (**C**). Light quality effects on H_2_O_2_ content (**B**) and MDA content (**D**). Different uppercase letters indicate significant differences among time points within the same treatment, and different lowercase letters indicate significant differences among treatments at the same time point (*p* < 0.05; one-way ANOVA followed by Tukey’s HSD test).

Under different photoperiods, H_2_O_2_ content showed varied but generally modest temporal changes, and no intergroup differences were detected at any time point (Figure 4A). MDA content decreased significantly on day 3 across all groups (*p* < 0.05) and then stabilized, again with no intergroup differences throughout the cultivation period (Figure 4C).

Under different light qualities, H_2_O_2_ responses diverged among treatments: white light decreased on day 3 and then recovered, blue light remained stable, and red light increased transiently on days 3–7 before returning to baseline (Figure 4B). Red light showed significantly lower H_2_O_2_ than white light on days 1 and 15 (*p* < 0.05). MDA patterns were similar across all light qualities, with significant decreases on day 3 (*p* < 0.05) and subsequent stabilization (Figure 4D). Blue light exhibited significantly lower initial MDA than white light on day 1 (*p* < 0.05), with no differences at later time points.

#### 2.1.5. Phlorotannin Content

**Figure 5:**
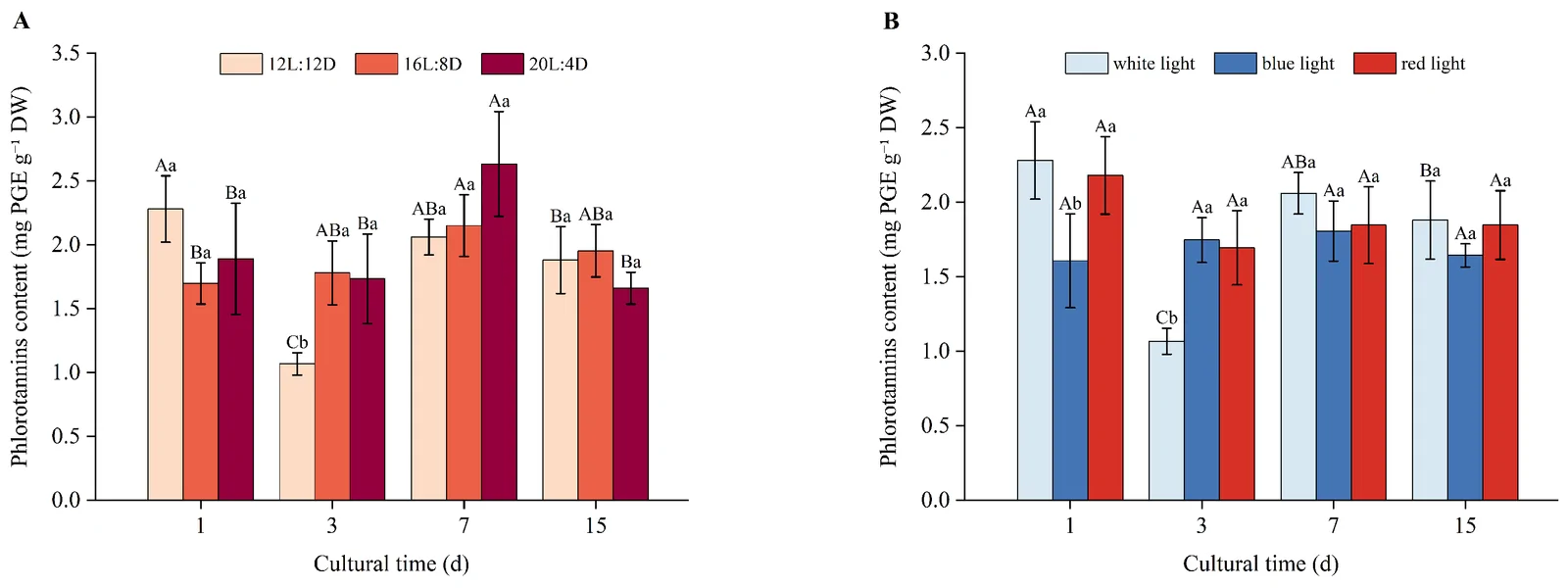
Effects of photoperiod and light quality on phlorotannin content in *Sargassum muticum* during 15 days of cultivation: (**A**) Photoperiod treatments. (**B**) Light quality treatments. Different uppercase letters indicate significant differences among time points within the same treatment, and different lowercase letters indicate significant differences among treatments at the same time point (*p* < 0.05; one-way ANOVA followed by Tukey’s HSD test).

Phlorotannin content showed time-dependent responses to photoperiod (Figure 5A). Values in the 16L:8D and 20L:4D groups peaked on day 7 and subsequently decreased. In contrast, the 12L:12D group showed a marked decrease on day 3, followed by partial recovery on day 7. On day 7, the value in the 12L:12D group was significantly lower than those in the 16L:8D and 20L:4D groups (*p* < 0.05). By day 15, no significant differences were detected among the three groups.

Under different light qualities (Figure 5B), white light induced a significant decrease on day 3 (*p* < 0.05), after which levels stabilized. The blue- and red-light groups maintained relatively stable phlorotannin content throughout the cultivation period (*p* > 0.05). On day 1, blue light was significantly lower than white and red light (*p* < 0.05); on day 3, the pattern reversed, with white light significantly lower than both blue and red light (*p* < 0.05). No intergroup differences were observed on days 7 and 15.

### 2.2. Transcriptomic Responses

Transcriptome sequencing was performed on 12 samples from four treatment groups: white light (12L:12D), long photoperiod (20L:4D), blue light, and red light, collected on day 7, a time point identified by time-course physiological monitoring as representing a key phase of light-responsive physiological adjustment (see Section 4.7 for detailed rationale). In total, 573.4 million high-quality reads were obtained, averaging 47.8 million clean reads per sample. Across all samples, the percentage of bases with Q30 exceeded 94%, indicating high sequencing quality. De novo assembly of all clean reads using Trinity yielded 73,091 unigenes, of which 73,048 were expressed. Principal component analysis (PCA) revealed clear separation among treatment groups at the transcriptomic level.

**Figure 6:**
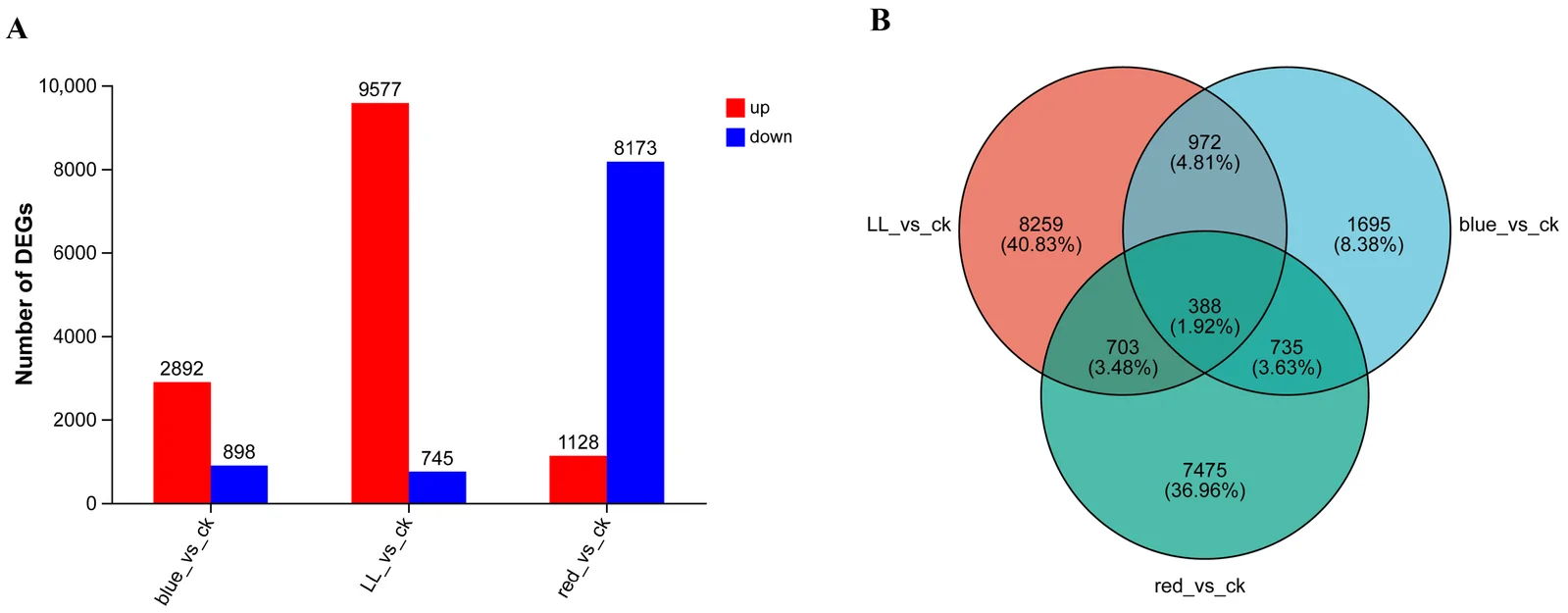
DEGs identification and overlap analysis: (**A**) DEG counts for three treatments. Differential expression analysis was performed between three light treatment groups and the white light control (CK) using DESeq2. DEGs were identified with the criteria of *p* < 0.05 and |log_2_FC| ≥ 2. (**B**) Venn diagram of DEGs. The blue-light group (blue circle), extended photoperiod (LL) group (orange circle), and red-light group (green circle) each display their unique DEGs.

Using white light (12L:12D) as the control, we identified DEGs in long photoperiod (20L:4D), blue light, and red light treatments (Figure 6A, Supplementary Figure S1). Long photoperiod showed the largest transcriptomic response, with 10,322 DEGs that were predominantly upregulated (92.8%). Blue light induced 3790 DEGs (76.3% upregulated). Red light showed 9301 DEGs but with predominantly downregulated expression (87.9%), contrasting sharply with the other treatments. Venn diagram analysis (Figure 6B) revealed 388 shared core light-responsive genes, with most DEGs being treatment-specific; detailed counts for each subset are shown in Figure 6B.

Among the 73,091 assembled unigenes, functional annotation identified nine cryptochrome-like and four phytochrome-like genes; notably, no aureochrome homologs were detected. Four cryptochrome-like and two phytochrome-like genes were differentially expressed across light treatments, exhibiting two contrasting patterns. TRINITY_DN16746_c1_g1 and TRINITY_DN11397_c0_g1 were most highly expressed under control conditions and markedly suppressed under red light (↓81% and ↓79% relative to CK, respectively; *p* < 0.01), with little response to blue light, whereas TRINITY_DN11226_c0_g2 and TRINITY_DN67347_c0_g1 were significantly upregulated under red light (4.9-fold and 8.0-fold relative to CK, respectively). The two differentially expressed phytochrome-like genes showed a similarly divergent pattern: TRINITY_DN16274_c0_g3 was highly expressed under CK and downregulated under red light (↓82%), while TRINITY_DN199997_c0_g1 was strongly induced under red light (9.7-fold relative to CK). Among the 388 core light-responsive DEGs, two transcription factor genes were identified, belonging to the bZIP and MYB families, respectively.

GO enrichment analysis (Supplementary Figure S2) revealed distinct functional profiles. Long photoperiod (680 terms) enriched catalytic activity and small-molecule binding (MF); small-molecule metabolic processes, MAPK cascade, and amino acid metabolism (BP); and cellular anatomical entity (CC). Blue light (552 terms) enriched chlorophyll binding and tetrapyrrole binding (MF); photosynthesis, light harvesting in photosystem I, and energy generation (BP); and photosystem I/II (CC). Red light (712 terms) enriched catalytic activity on DNA/nucleic acids (MF); DNA metabolic process and calcium ion import (BP); and cellular anatomical entity, membrane, and chloroplast envelope (CC).

**Figure 7:**
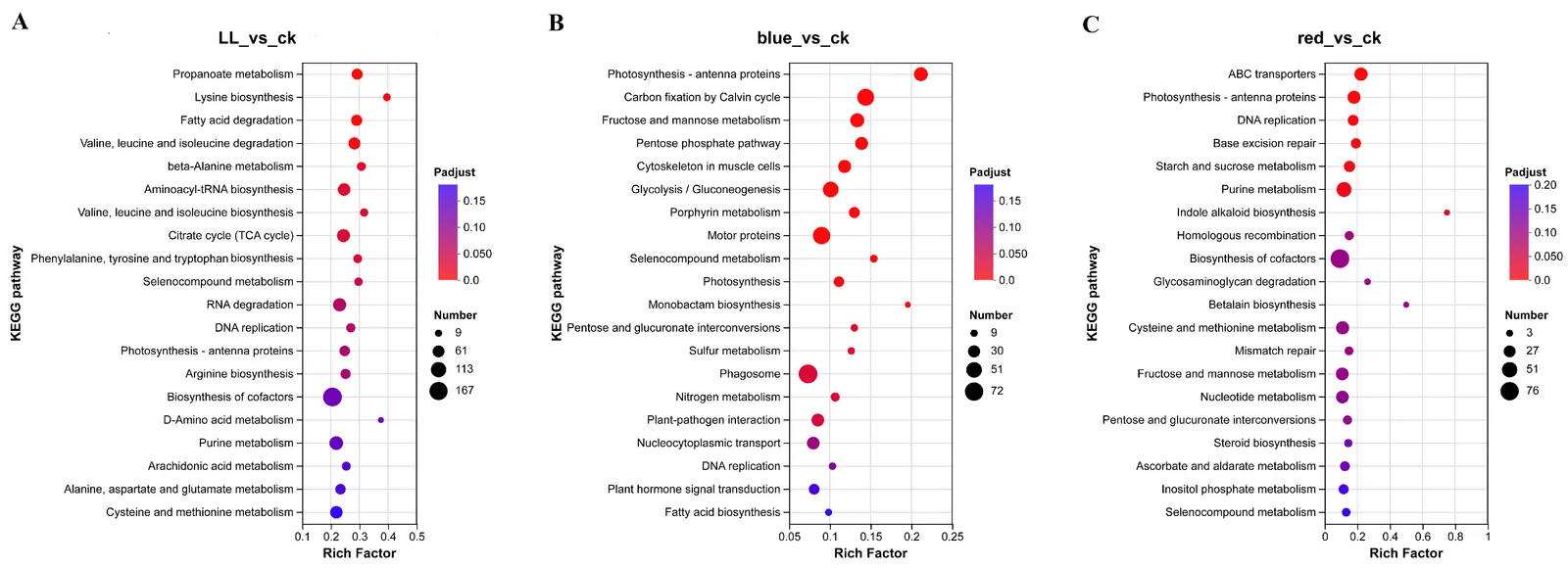
KEGG pathway enrichment analysis of DEGs under different light regimes. The top 20 significantly enriched KEGG pathways are shown. The *y*-axis lists pathway names; the *x*-axis shows the Rich Factor. Bubble size reflects the number of enriched DEGs, and bubble color indicates the adjusted *p*-value (Padjust), with redder colors denoting higher significance. (**A**) Enriched pathways in the LL_vs_ck comparison. (**B**) Enriched pathways in the blue_vs_ck comparison. (**C**) Enriched pathways in the red_vs_ck comparison.

KEGG pathway enrichment (Figure 7) showed treatment-specific enrichment patterns. Long photoperiod enriched 128 pathways, including propanoate metabolism, lysine biosynthesis, and fatty acid degradation. Blue light enriched 119 pathways, including photosynthesis-antenna proteins, carbon fixation in photosynthetic organisms, and fructose and mannose metabolism. Red light enriched 128 pathways, including ABC transporters, photosynthesis-antenna proteins, and DNA replication.

### 2.3. Metabolomic Responses

Untargeted LC-MS metabolomics yielded 949 putatively annotated metabolites across all twelve samples, of which a subset was mapped to 86 KEGG pathways. Principal component analysis (PCA) revealed clear separation among the four treatment groups (PC1: 22.80%; PC2: 18.90%), with tight within-group clustering (Supplementary Figure S3), indicating treatment-specific metabolic profiles and data reproducibility.

Differential metabolite analysis (VIP > 1, *p* < 0.05) detected 154 (red light), 120 (blue light), and 60 (long photoperiod) significantly altered metabolites relative to the white-light control (Supplementary Figures S4 and S5). Venn diagram analysis revealed that 12 putatively annotated metabolites were shared among the three comparisons. Because the assignments were not verified using authenticated standards, the reported compound names should be interpreted as tentative annotations rather than confirmed chemical identifications.

**Figure 8:**
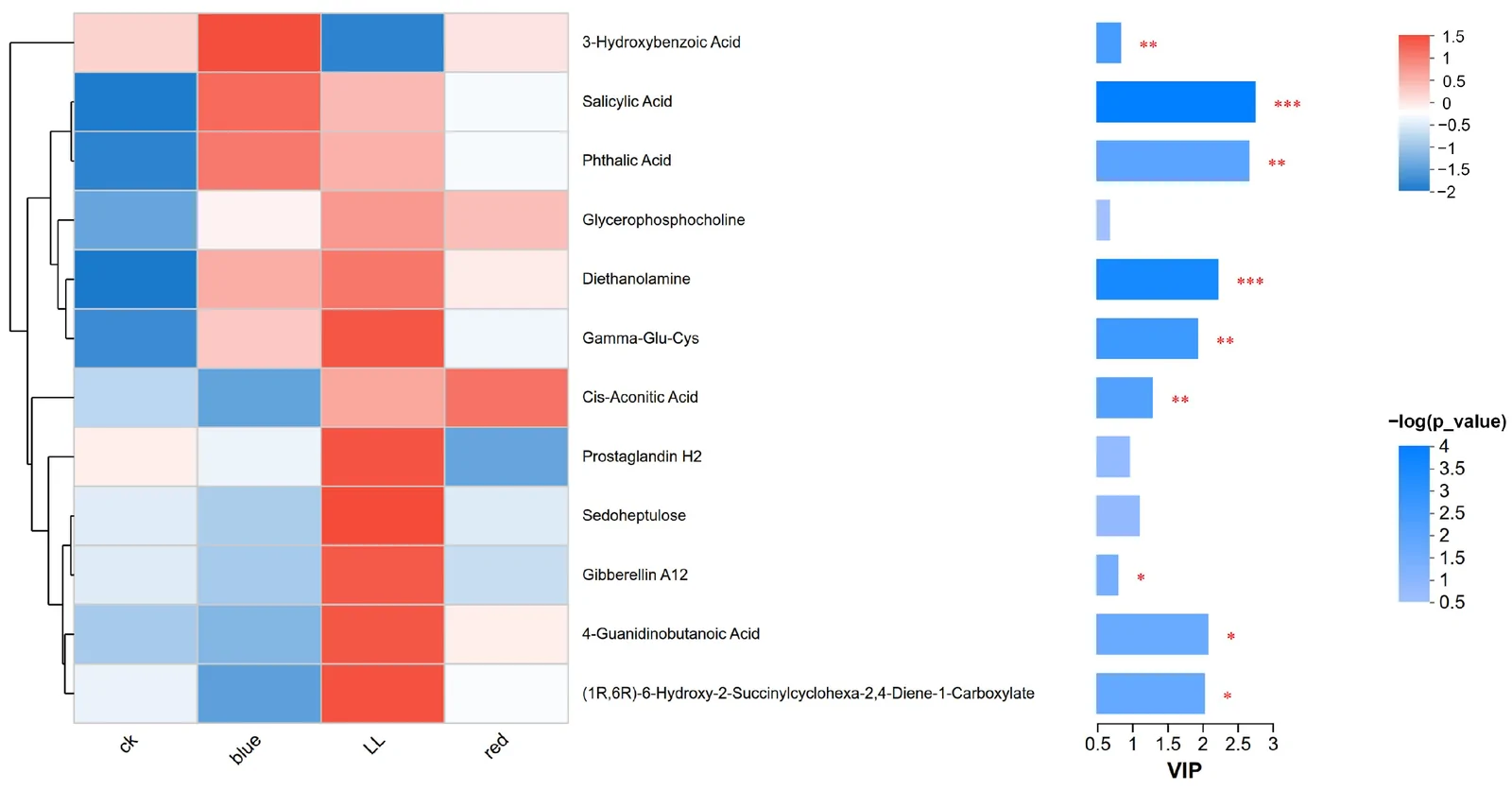
Hierarchical clustering heatmap of 12 core differential metabolites across four light treatments (CK, blue, LL, and red). The color scale represents log_2_ fold changes relative to the control, ranging from −2 to 1.5. Adjacent bars show PLS-DA VIP scores, with bar color corresponding to −log_10_ *p*-value. Asterisks denote significance levels: * *p* < 0.05; ** *p* < 0.01; *** *p* < 0.001.

PLS-DA-based variable importance in projection (VIP) analysis identified salicylic acid (VIP = 2.8), diethanolamine (VIP = 2.5), and phthalic acid (VIP = 2.3) as the most discriminatory metabolites distinguishing light-quality treatments from control (Figure 8). Features putatively annotated as salicylic acid, γ-glutamylcysteine, and cis-aconitic acid showed consistently higher relative abundance under the three light treatments. These recurrent changes identify candidate metabolic responses for future validation but do not establish regulatory functions for the annotated compounds. Hierarchical clustering separated these features according to their treatment-dependent abundance patterns. Because phthalic acid and dibutyl phthalate may originate from laboratory materials, they were excluded from mechanistic interpretation.

KEGG pathway enrichment analysis (Supplementary Figure S6) revealed that differential metabolites were significantly enriched in 47 metabolic pathways (*p* < 0.05), predominantly involving carbohydrate metabolism (pentose phosphate pathway, galactose metabolism), lipid biosynthesis (cutin, suberine, and wax biosynthesis), and aromatic amino acid pathways (tyrosine metabolism, phenylalanine/tyrosine/tryptophan biosynthesis, and beta-alanine metabolism). Hierarchical clustering of the top 50 differential metabolites (Supplementary Figure S7) identified five distinct subclusters exhibiting pronounced treatment-specific expression patterns, further supporting the light-quality-specific metabolic adjustments observed in *S. muticum*.

### 2.4. Integrated Transcriptomic–Metabolomic Correlation Analysis

#### 2.4.1. Correlation Analysis Between Differentially Expressed Genes and Metabolites

**Figure 9:**
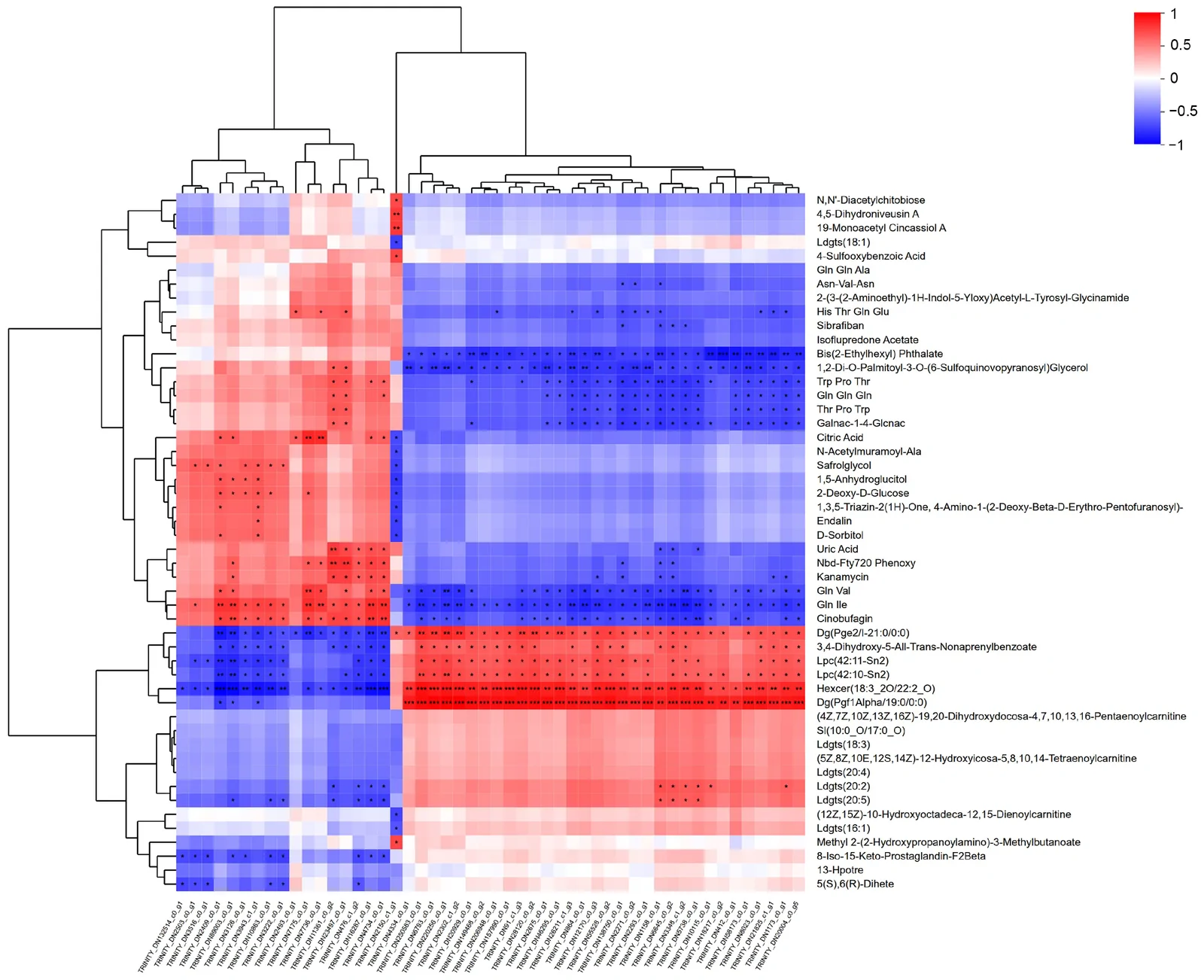
Pearson correlation heatmap between differentially expressed genes (*x*-axis) and the core differential metabolites (*y*-axis). The color scale represents correlation coefficients from −1 (blue) to +1 (red). Hierarchical clustering was performed using Euclidean distance. Asterisks denote the level of statistical significance of the correlation: * indicates *p* < 0.05, ** indicates *p* < 0.01, and *** indicates *p* < 0.001.

Pearson correlation analysis between 388 shared DEGs and differential metabolites revealed three distinct clusters (Figure 9). Category I comprised negatively correlated metabolites (r < −0.8, *p* < 0.01), including amino acids (glutamate, glutamine, and aspartate) and sugar derivatives. Category II consisted of strongly positively correlated metabolites (r > 0.8, *p* < 0.01), predominantly lipids (lysophosphatidylcholines, hexosylceramides). Category III included weakly correlated metabolites (|r| < 0.3), such as N,N′-diacetylchitobiose.

To refine pathway-level associations, we analyzed correlations between KEGG-annotated genes (*n* = 79) and metabolites (*n* = 36) (Figure S8). Strong positive correlations were observed for features putatively annotated as cucurbitacin D, urocanic acid, xanthine, adenine, and gallocatechin. These correlations describe statistical covariation only and do not confirm compound identity, pathway membership, or regulatory relationships. Conversely, TCA cycle intermediates (citric acid, cis-aconitic acid), folate derivatives (tetrahydrofolyl diglutamate), and amino acid metabolites (L-2-aminoethyl seryl phosphate) exhibited strong negative correlations (r < −0.7, *p* < 0.01). Fatty acid metabolites showed weak or no correlations, suggesting that their variation was not strongly linked to the expression of the analyzed KEGG-annotated genes.

#### 2.4.2. Putatively Annotated Phenolic Metabolites and Expression of Candidate PKS and ACC Transcripts

Untargeted metabolomic analysis yielded putative Level 2 annotations for several phenolic and aromatic compounds. A feature putatively annotated as phloroglucinol, the monomeric building block of phlorotannins, was detected in all treatments but did not differ significantly among treatments (|log_2_FC| < 1, *p* > 0.05). Features putatively annotated as salicylic acid, 3-hydroxybenzoic acid, phthalic acid, and gallocatechin were also detected. However, these compounds are not established intermediates of the brown-algal phlorotannin biosynthetic pathway and are therefore considered here only as tentative components of the broader metabolic response to light. Salicylic acid showed increased relative abundance under the three light treatments, whereas gallocatechin showed decreased relative abundance under red light.

**Figure 10:**
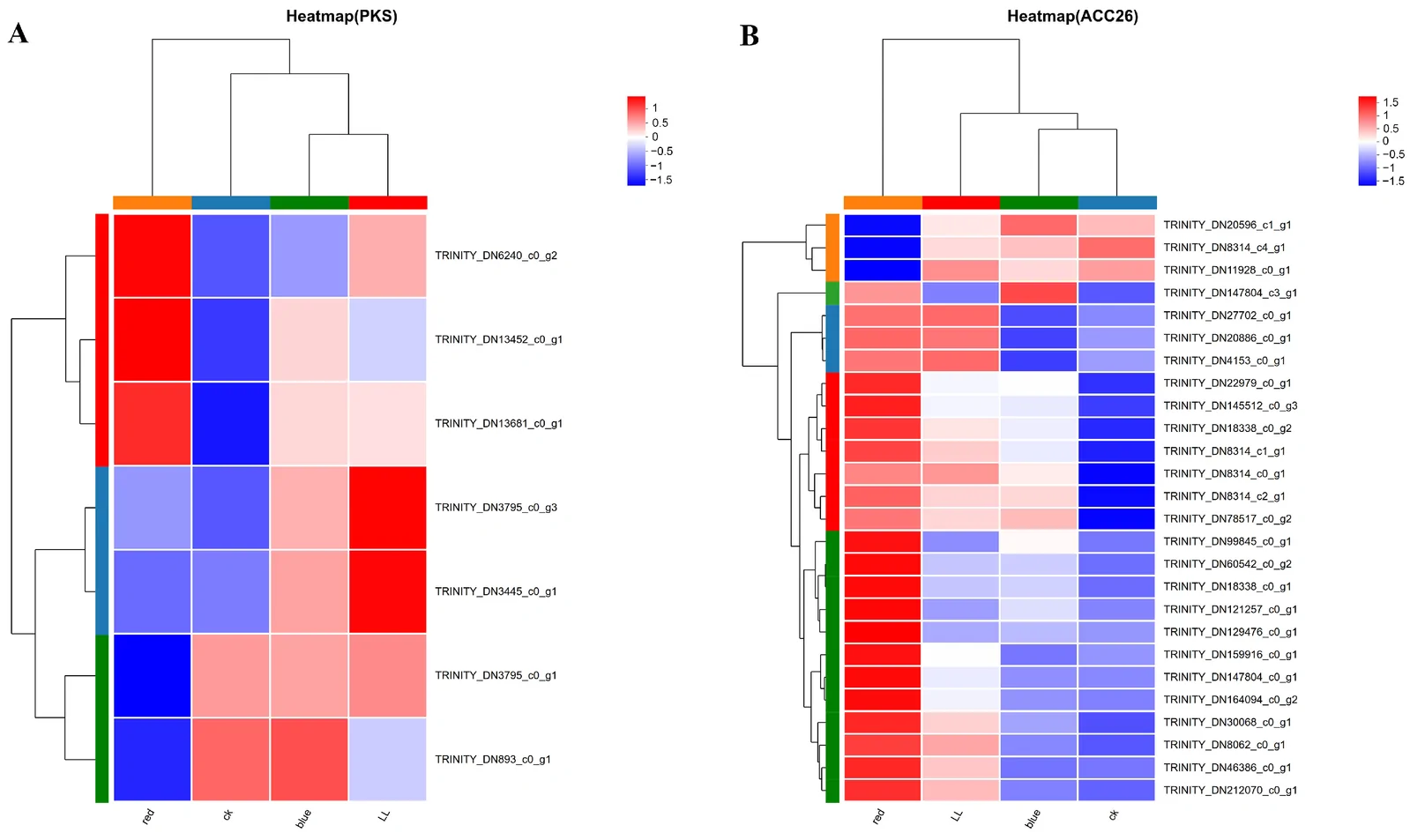
Expression profiles of candidate PKS- and ACC-related transcripts under different light treatments: (**A**) Heatmap of seven putatively annotated PKS-related transcripts. (**B**) Heatmap of 26 putatively annotated ACC-related transcripts. Gene expression (log2-FPKM) was Z-score normalized across samples and clustered using Euclidean distance and complete linkage. The color scale represents normalized expression levels from −1.5 (blue, downregulated) to +1.5 (red, upregulated). The colored bars above the heatmaps indicate sample groupings by treatment, while the colored bars to the left of the heatmaps indicate distinct gene clusters.

To identify transcriptional responses potentially associated with polyketide precursor metabolism, we examined transcripts putatively annotated as PKS and ACC candidates (Figure 10). Seven transcripts were putatively annotated as PKS-related sequences, including four candidate type III PKS transcripts. Type III PKSs have been implicated in phloroglucinol formation in other brown algae, but the biochemical functions of the transcripts identified here were not experimentally validated. Hierarchical clustering separated the seven candidate transcripts into two groups with contrasting expression patterns (Figure 10A). At the sample level, blue light and extended photoperiod shared the most similar expression profiles, while red light was the most divergent condition. Cluster I comprised three genes (TRINITY_DN6240_c0_g2, TRINITY_DN13452_c0_g1, and TRINITY_DN13681_c0_g1) that were markedly upregulated under red light, with an expression gradient of red > LL ≈ blue > ck. Cluster II contained four genes (TRINITY_DN3795_c0_g3, TRINITY_DN3445_c0_g1, TRINITY_DN3795_c0_g1, and TRINITY_DN893_c0_g1) that displayed the opposite pattern, with the highest expression under LL and the lowest under red light (LL > blue > ck > red). These contrasting patterns indicate that different light treatments were associated with differential expression of putative PKS-related transcripts; however, they do not demonstrate differential enzymatic activity or phloroglucinol flux.

Twenty-six transcripts were putatively annotated as ACC-related sequences. ACC catalyzes malonyl-CoA formation, but malonyl-CoA is shared by fatty acid synthesis and several other metabolic pathways. Consequently, the expression of these candidate transcripts cannot be assigned specifically to phlorotannin biosynthesis. Hierarchical clustering showed that red light produced the most distinct ACC expression profile, while blue light and the control were most similar, with LL intermediate (Figure 10B). The 26 ACC genes were grouped into two major clusters. Cluster I exhibited heterogeneous expression patterns; notably, three genes (TRINITY_DN20596_c1_g1, TRINITY_DN8314_c4_g1, and TRINITY_DN11928_c0_g1) were highly expressed under control and LL conditions but substantially downregulated under red light. Cluster II displayed a coherent expression gradient of red > LL > blue > ck, with the majority of genes showing strong upregulation under red light and low expression under control conditions. These results show a bidirectional transcriptional response among the putative ACC-related transcripts under red light, with most candidates showing increased expression and a smaller subset showing decreased expression.

## 3. Discussion

### 3.1. Differentiated Responses to Light Environment: Growth and Photosynthetic Physiology

Light conditions critically regulate macroalgal growth and photosynthetic performance through light intensity, photoperiod, and spectral composition. Regarding photoperiod, studies demonstrate that illumination systems incorporating dark periods can enhance productivity by improving light absorption efficiency [31]. Responses to light quality are notably species-specific; for instance, blue light stimulated growth in the green alga *Ulva australis* (Chlorophyta) [32], whereas the red alga *Neopyropia leucosticta* (formerly *Porphyra leucosticta*) (Rhodophyta) demonstrated reduced photosynthetic capacity under blue light but enhanced performance under red and white light [33]. Furthermore, recent findings support the notion that red light generally enhances biomass productivity, while blue light promotes protein production in microalgae [34].

In the present study, extended photoperiod (20L:4D) promoted growth and was associated with a transient increase in phlorotannin content but reduced pigment contents, which may reflect a growth-oriented response accompanied by photoacclimation. Blue-light treatment suppressed growth but sustained higher chlorophyll *a* content and stable *F*v/*F*m and Y(II) values, suggesting prioritization of photochemical efficiency and photoprotection over biomass accumulation. Red light did not confer growth advantages over white light, yet it induced pronounced transcriptional repression accompanied by morphological changes (longer branches, thinner blades), implying potential rearrangements in energy utilization and photomorphogenesis. The pigment decline under extended photoperiod may reflect biomass dilution due to rapid growth and antenna size adjustment during photoacclimation. The stability of *F*v/*F*m and Y(II) under blue light suggests reduced PSII damage or enhanced repair capacity. In brown algae, decreased *F*v/*F*m typically indicates PSII photoinhibition, while elevated NPQ reflects thermal dissipation of excess energy to reduce ROS pressure [35]. Photoinhibition occurs at high irradiance in *S. muticum*, indicating that growth enhancement and photoprotection often exhibit condition-dependent trade-offs.

Together, these findings point to differentiated response patterns: extended photoperiod appeared to favor biomass accumulation, whereas blue light appeared to prioritize photosynthetic efficiency at the expense of growth. Such response differences may also be linked to distinct patterns of secondary metabolite accumulation.

### 3.2. Relationships Between Oxidative Stress Markers and Phlorotannin Content

Phlorotannins are widely recognized for their antioxidant and ultraviolet-protective ecological functions, and their accumulation can be influenced by light, desiccation, and nutrient availability [36]. Phlorotannins have been shown to exhibit antioxidant activity, attributed to their polyphenolic structure and ROS-scavenging capacity.

Under blue light, oxidative damage markers remained relatively low while phlorotannin content showed limited temporal variation. Under extended photoperiod, the transiently higher phenolic-equivalent values were not accompanied by corresponding increases in H_2_O_2_ or MDA. These patterns indicate an association between the bulk phenolic response and cellular redox status. However, because individual phlorotannins and the antioxidant activity of the extracts were not directly characterized, the present data cannot establish that phlorotannins were responsible for the observed oxidative-stress patterns.

The question of constitutive versus inducible phlorotannin accumulation remains debated. The LC–MS feature putatively annotated as phloroglucinol did not differ significantly among treatments. Because this annotation was not confirmed using an authenticated standard and the colorimetric assay did not specifically quantify phlorotannins, no conclusion can be drawn regarding whether light affected phloroglucinol synthesis, downstream polymerization, or other components of the extractable phenolic pool.

### 3.3. Light-Responsive Molecular Network Revealed by Transcriptional Reprogramming

Transcriptomic analysis provides critical insights into the molecular mechanisms underlying algal light responses. Transcriptomic studies in other algae have revealed extensive light-responsive gene regulation across different wavelengths [37].

In the present study, transcriptional reprogramming induced by different light conditions exhibited pronounced differentiation in scale and directionality. Extended photoperiod (20L:4D) induced 10,322 DEGs with predominantly upregulated expression (92.8%); blue light induced 3790 DEGs with 76.3% upregulation; and red light induced 9301 DEGs but with predominantly downregulated expression (87.9%). This pattern suggests that *S. muticum* exhibits light quality- and photoperiod-specific global transcriptional responses, which may be associated with adjustments in resource allocation and stress-related processes. The predominantly upregulated expression pattern (92.8%) under extended photoperiod may indicate a general activation of transcriptional activity. In contrast, the predominantly downregulated pattern (87.9%) under red light is notable and may reflect physiological constraints on growth or altered photosystem energy distribution, potentially contributing to the broad transcriptional repression observed.

The 388 core light-responsive genes identified across all treatments may represent conserved regulatory nodes in the light response network, potentially amplifying signals through transcription factor hierarchies rather than acting directly as metabolic enzymes, which could help explain the pronounced differences in DEG numbers and directionality among light treatments. Consistent with this, cryptochrome-like and phytochrome-like genes were identified among the differentially expressed transcripts, providing preliminary evidence for canonical photoreceptor involvement in the light response of *S. muticum*. Notably, no aureochrome homologs were detected despite their recognized role as blue-light photoreceptors in stramenopiles [38], possibly reflecting limitations of de novo assembly and annotation sensitivity rather than genuine gene loss. Red light was the most prominent modulator of photoreceptor gene expression, significantly affecting all six differentially expressed photoreceptor genes, whereas blue light had comparatively modest effects. The divergent expression patterns suggest functionally distinct photoreceptor subfamilies, although transcript abundance does not necessarily reflect photoperception activity. The identification of bZIP and MYB transcription factors among the 388 core light-responsive DEGs, together with the significant enrichment of MAPK cascade components under extended photoperiod, further suggests that light signals may be transduced through phosphorylation cascades to downstream transcriptional effectors.

### 3.4. Metabolic Responses to Altered Light Regimes

Untargeted metabolomics detected 60–154 differentially abundant, putatively annotated metabolites across the different comparisons, including 12 annotations shared among the three light treatments. These changes represent candidate metabolic responses rather than demonstrated regulatory nodes. Dibutyl phthalate and phthalic acid are common plasticizer-associated contaminants in LC–MS analyses [39]. Their signals may partly or wholly reflect contamination from laboratory materials; therefore, both compounds were excluded from mechanistic interpretation.

A feature putatively annotated as salicylic acid showed increased relative abundance under all three light treatments. Although salicylic acid participates in stress signaling in model plants [40], its identity was not confirmed using an authenticated standard, and its function in brown algae remains poorly characterized. This annotation should therefore be regarded as a candidate observation requiring targeted chemical and functional validation. The consistent accumulation of γ-glutamylcysteine across all treatments is particularly noteworthy. As γ-glutamylcysteine is a direct precursor of glutathione, its accumulation may indicate an enhanced demand for glutathione biosynthesis, which could be related to increased cellular thiol-redox buffering requirements under altered light conditions.

Correlation analysis between metabolites and the 388 core light-responsive genes revealed distinct clustering patterns: amino acids and sugar derivatives predominantly showed negative correlations with G388 (r < −0.8, *p* < 0.01), whereas lipid metabolites, including lysophosphatidylcholines and hexosylceramides, exhibited strong positive correlations (r > 0.8, *p* < 0.01). These coordinated patterns are consistent with treatment-associated changes among amino acid, carbohydrate, and lipid metabolism. However, correlation analysis cannot determine the direction of metabolic flux or demonstrate resource reallocation toward phlorotannin biosynthesis. Direct verification would require targeted enzyme activity assays, ^13^C-isotope-based metabolic flux analysis, and proteomic quantification, which we propose as essential next steps. Nevertheless, such correlation-based multi-omics integration remains a widely adopted hypothesis-generating strategy in non-model organisms lacking established genetic tools [41].

KEGG enrichment highlighted treatment-associated changes in carbohydrate, lipid-related, and aromatic amino acid pathways. These results provide a broad overview of metabolic reorganization but do not establish flux toward a specific phenolic biosynthetic pathway.

### 3.5. Light-Dependent Expression of Candidate PKS and ACC Transcripts

The transcriptome contained seven transcripts putatively annotated as PKS-related sequences, including four candidate type III PKS transcripts, together with 26 putative ACC-related transcripts. These candidates exhibited light quality- and photoperiod-dependent expression patterns. Type III PKSs have been associated with phloroglucinol formation in other brown algae, but the functions of the transcripts identified in *S. muticum* were not verified using heterologous expression, enzyme assays, or genetic approaches. Their expression patterns should therefore be interpreted as candidate molecular responses rather than direct evidence of altered phlorotannin biosynthesis.

ACC catalyzes the formation of malonyl-CoA, a precursor shared by fatty-acid synthesis and polyketide metabolism. Three putative ACC-related transcripts showed increased expression under extended photoperiod, coinciding with increased growth and a transient increase in phlorotannin content. However, this concurrence does not demonstrate that the additional malonyl-CoA was directed toward phenolic synthesis. The expression response may instead reflect broader changes in membrane-lipid synthesis, growth, or carbon metabolism.

## 4. Materials and Methods

### 4.1. Algal Material and Experimental Design

Specimens of *S. muticum* were collected at low tide from a coastal site in Rongcheng, Shandong Province, China (37°25′ N, 122°59′ E). After collection, samples were placed in insulated containers filled with seawater and kept at 4 °C during transport to the laboratory. Upon arrival, samples were rinsed with filtered seawater (FSW, 0.80 μm) to remove surface fouling and were then acclimated for 5 days in 40 L glass aquaria supplied with a recirculating natural-seawater system and continuous ambient-air aeration. Acclimation conditions were temperature 15 ± 1 °C, salinity 30 ± 1, light intensity 100 ± 10 μmol photons m^−2^ s^−1^, and photoperiod 12L:12D.

Experiment I—photoperiod treatments: Three photoperiod treatments were established, each with three biological replicates (*n* = 3): (1) reference photoperiod (CK), 12L:12D; (2) medium photoperiod (ML), 16L:8D; and (3) long photoperiod (LL), 20L:4D. Cultures were maintained under white light at 100 ± 5 μmol photons m^−2^ s^−1^ and 15 ± 0.5 °C for 15 d. The 12L:12D condition was adopted as the reference following convention in temperate macroalgal photoperiod studies; accordingly, “CK” denotes a reference/baseline condition rather than an untreated control.

Experiment II—light-quality treatments: Three light-quality treatments were established, each with three biological replicates (*n* = 3): (1) white light (WL, reference; ~6500 K, continuous emission 400–760 nm); (2) blue light (BL, peak 450–475 nm); and (3) red light (RL, peak 625–650 nm). Photoperiod, light intensity, temperature, and experimental duration were identical to Experiment I. Spectral parameters were based on the manufacturer’s technical specifications; independent spectroradiometric verification was not performed. Photon flux density at the flask water surface was verified with a calibrated quantum sensor and adjusted to 100 ± 5 μmol photons m^−2^ s^−1^ by regulating LED-panel–flask distance and dimmer output.

Sampling design and tissue processing: A destructive sampling strategy was employed in both experiments. For each treatment, 12 flasks were prepared at the start of the experiment, each containing 5.0 ± 0.2 g fresh weight of *S. muticum* apical branches and serving as one independent biological replicate. At each of the four sampling time points (days 1, 3, 7, and 15), three flasks per treatment were harvested in their entirety and subsequently terminated; the remaining flasks continued under the assigned treatment conditions until their designated harvest date. No repeated sampling was performed on the same flask or thallus, ensuring that measurements at each time point were derived from three fully independent biological replicates (*n* = 3). At each harvest, fresh weight was recorded, and approximately 2-cm apical segments were collected for chlorophyll fluorescence measurements. Fresh tissue (~1.0 g) was flash-frozen in liquid nitrogen and stored at −80 °C for RNA extraction, metabolomics analysis, and biochemical assays. The remaining tissue was freeze-dried, ground to a 60-mesh powder, and stored at −20 °C for phlorotannin quantification.

### 4.2. Measurement of Relative Growth Rate

The relative growth rate (RGR) was determined using the fresh-weight method. At each sampling, thalli were gently removed, surface moisture was blotted with filter paper, and fresh weight was measured immediately. RGR was calculated as

$$\mathrm{RGR}(\%\cdot d^{-1})=\frac{(\ln Wt-\ln W0)\times 100}{∆t}$$

where *Wt* is the fresh weight at time t (g), *W*0 is the initial fresh weight (g), Δ*t* is the interval duration (d), and ln denotes the natural logarithm.

### 4.3. Photosynthetic Pigment Quantification

Accurately weigh 0.1 g fresh algal tissue, add liquid nitrogen, and grind thoroughly to a powder. Quickly transfer the powder to a 10 mL centrifuge tube, add 1.5 mL pre-chilled 80% acetone (*v*/*v*; acetone–double-distilled water = 80:20), and vortex immediately for 30 s. Tubes were wrapped in aluminum foil and kept at 4 °C in the dark for 24 h. After extraction, samples were centrifuged at ~4000× *g* for 10 min at 4 °C. The supernatant was carefully transferred to a new tube. An aliquot of 0.2 mL of supernatant was diluted to 1.0 mL with 80% acetone (5-fold dilution). Absorbance at 664, 630, 510, and 480 nm was measured using a microplate reader (VICTOR NIVO 5S; PerkinElmer, Shelton, CT, USA). Each sample was measured in triplicate (technical replicates), and the mean was used. An 80% acetone solution served as the blank. Pigment content was calculated following Jeffrey and Humphrey [42] and Wellburn [43] using the following equations:

$$Chl\text{-}a=(11.47{OD}_{664}-0.40{OD}_{630})\times V\times D/W/1000$$

$$Chl\text{-}c=(24.36{OD}_{630}-3.73{OD}_{664})\times V\times D/W/1000$$

$$Car=7.6\times ({OD}_{480}-1.49\times {OD}_{510})\times V\times D/W/1000$$

where pigment content is expressed as mg g^−1^ FW (fresh weight), *V* is the total volume of extract (mL), *W* is the sample fresh weight (g), and *D* is the dilution factor.

### 4.4. Determination of Chlorophyll Fluorescence Parameters

Chlorophyll fluorescence parameters of photosystem II (PSII) were measured using a pulse-amplitude modulated fluorometer (Water-PAM; Walz, Effeltrich, Germany). Apical thallus segments (~2 cm) were dark-adapted for 15 min prior to measurement. The maximum quantum yield of PSII photochemistry (*F*v/*F*m) was recorded first. Subsequently, a rapid light curve (RLC) protocol was applied to determine the effective quantum yield [Y(II)], photochemical quenching (qP), and non-photochemical quenching (NPQ). During RLC acquisition, actinic light was applied at nine incremental intensities (29 to 1253 μmol photons m^−2^ s^−1^), with 10 s exposure at each step. Instrument settings were as follows: measuring light, 0.1 μmol photons m^−2^ s^−1^; saturating pulse, 87 μmol photons m^−2^ s^−1^ with 0.8 s pulse duration. Each sample was measured 3–5 times, and the mean value was used for analysis.

### 4.5. Determination of Oxidative Stress Indicators

Fresh algal tissue (0.1 g) was weighed and mixed with 1.0 mL ice-cold phosphate-buffered saline (PBS, 0.1 M, pH 7.4). The mixture was kept on ice and homogenized for 2–3 min at 4 °C using a tissue grinder (Tissuelyser-24; Jingxin, Shanghai, China) until uniform. After homogenization, the sample was centrifuged at 13,400× *g* for 10 min at 4 °C. We then carefully collected the supernatant, avoiding both the lipid layer and pellet. The supernatant was transferred to a pre-chilled centrifuge tube and stored on ice for subsequent assays.

Hydrogen peroxide (H_2_O_2_) and malondialdehyde (MDA) contents were determined using commercial kits (Nanjing Jiancheng Bioengineering Institute, Nanjing, China). H_2_O_2_ was assayed by the ammonium molybdate colorimetric (molybdenum Blue) method: under acidic conditions, H_2_O_2_ reacts with ammonium molybdate to form a yellow peroxomolybdate complex with characteristic absorbance at 405 nm. MDA was measured by the thiobarbituric acid (TBA) colorimetric method: under high-temperature, acidic conditions, the lipid peroxidation product MDA condenses with TBA to form a pink MDA TBA adduct with maximal absorbance at 532 nm.

### 4.6. Determination of Phlorotannin Content

Phlorotannin content was determined using the Folin–Ciocalteu colorimetric method as described by Yan et al. [44]. Freeze-dried algal powder (0.2 g) was extracted with 3.0 mL of 15% (*v*/*v*) ethanol at 4 °C for 24 h. The samples were briefly subjected to microwave treatment and then centrifuged. The supernatant was collected for analysis. An aliquot of the supernatant (50 μL) was mixed with 650 μL of water and 100 μL of Folin–Ciocalteu reagent, followed by the addition of 200 μL of 20% Na_2_CO_3_. The mixture was incubated in darkness for 3 h, and absorbance was measured at 710 nm.

A calibration curve was prepared using phloroglucinol at serial concentrations. The calibration curve (R^2^ ≥ 0.999) was used to express the results as milligrams of phloroglucinol equivalents per gram of dry weight (mg PGE g^−1^ DW).

The Folin–Ciocalteu assay measures the reducing capacity of the extract and is not specific to phlorotannins. Co-extracted reducing substances may therefore contribute to the measured values [45]. Accordingly, the results are reported as phloroglucinol-equivalent total phenolic content and should not be interpreted as absolute concentrations of phlorotannins.

### 4.7. Sample Selection for Transcriptomic and Metabolomic Analyses

Day 7 was selected for transcriptomic and metabolomic analyses as a common sampling point representing an intermediate stage of physiological acclimation. At this time point, several growth, pigment, and photosynthetic variables showed treatment-related differences, and the photoperiod experiment showed pronounced differences in phlorotannin content. Although the light-quality treatments did not differ significantly in this phenolic index on day 7, use of a common sampling time enabled direct comparison of the broader molecular responses among the selected light regimes. For each treatment, three independent biological replicates (*n* = 3, corresponding to three independently harvested flasks) were subjected to both RNA sequencing and untargeted metabolomic profiling using aliquots of the same flash-frozen tissue.

### 4.8. RNA-Seq and Transcriptome Analysis

Total RNA was extracted using TRIzol reagent. RNA quality was assessed by spectrophotometry (OD260/280 = 1.8–2.1) and an Agilent 2100 bioanalyzer (Agilent Technologies, Santa Clara, CA, USA). Samples meeting quality criteria were used for library construction. Briefly, mRNA was enriched using oligo(dT) magnetic beads and then fragmented to approximately 300 bp. The fragmented mRNA was reverse-transcribed to synthesize cDNA. Sequencing libraries were subsequently prepared and sequenced on a DNBSEQ-T7 platform (PE150, ≥6 Gb per sample).

Raw reads were processed using fastp to remove adapters and filter out low-quality sequences. Clean reads were then subjected to de novo assembly using Trinity with a k-mer size of 25. The assembly was further optimized using TransRate, and redundant sequences were removed by clustering with CD-HIT to generate nonredundant transcripts. We evaluated assembly completeness using BUSCO. For functional annotation, nonredundant transcripts were searched against NR, Swiss-Prot, Pfam, COG, GO, and KEGG databases with an e-value cutoff of 1 × 10^−5^. Differentially expressed genes (DEGs) were identified using DESeq2, with thresholds of |log_2_FC| ≥ 2 and FDR < 0.05. We also performed GO and KEGG pathway enrichment analyses, and pathways with FDR < 0.05 were considered significantly enriched. All sequencing data have been deposited in the NCBI SRA database under accession number PRJNA1405268.

### 4.9. Untargeted Metabolomics Analysis

Tissue samples (approximately 50 mg) were homogenized in 400 μL extraction solvent (methanol–water = 4:1, *v*/*v*) at −10 °C and 50 Hz for 6 min. This was followed by ultrasound-assisted extraction at 5 °C for 30 min. Proteins were then precipitated at −20 °C, and the samples were centrifuged at 13,000× *g* for 15 min at 4 °C. The supernatant was collected for LC–MS analysis.

LC–MS analysis was performed on a UHPLC–Q Exactive HF-X system (Thermo Fisher Scientific, Bremen, Germany) equipped with an ACQUITY UPLC HSS T3 column. The mobile phase consisted of water/acetonitrile (A) and acetonitrile/isopropanol/water (B), both containing 0.1% formic acid. The flow rate was set at 0.40 mL min^−1^ with an injection volume of 3 μL. Electrospray ionization (ESI) was operated in both positive and negative modes, with capillary voltages of 3500 V and −3000 V, respectively. Full-scan MS1 data were acquired over an *m*/*z* range of 70–1050 at a resolution of 60,000 (FWHM at *m*/*z* 200). Data-dependent MS2 acquisition was triggered for the top 3 most intense precursor ions per scan cycle at a resolution of 7500, with collision energies of 20, 40, and 60 eV.

Metabolite annotation was performed using Progenesis QI software version 3.1 (Waters Corporation, Milford, MA, USA) by matching MS1 accurate mass (mass error < 10 ppm) and MS/MS fragmentation patterns against the HMDB, METLIN, and an in-house spectral library (Majorbio). Metabolite identifications in this study were classified as putative annotations (Level 2 according to the Metabolomics Standards Initiative [46]), based on spectral database matching without direct comparison to authenticated analytical standards. Features with more than 20% missing values within any group were removed, and the remaining missing values were imputed using the minimum value. Response intensities were normalized using total sum normalization, and variables with RSD > 30% in QC samples were excluded. The data were log_10_-transformed prior to statistical analysis.

### 4.10. Integrated Analysis of Transcriptomics and Metabolomics

To explore statistical associations between transcriptional and metabolic responses, differentially expressed transcripts and differentially abundant, putatively annotated metabolites were jointly mapped to KEGG pathways. Candidate transcripts annotated as PKS- or ACC-related sequences and selected phenolic or aromatic metabolite annotations were examined as a hypothesis-generating subset. Pearson correlation analysis was performed using SPSS Statistics 26.0 (IBM Corp., Armonk, NY, USA) to evaluate associations between the expression levels of DEGs and metabolite abundances (integrated peak areas). Correlations with r > 0.9 and *p* < 0.05 were considered statistically significant and retained for subsequent analysis.

## 5. Conclusions

This study characterized the physiological, transcriptomic, and metabolomic responses of *Sargassum muticum* to photoperiod extension and different light qualities. Extended photoperiod promoted growth and was associated with a transient increase in phlorotannin content and predominantly increased transcript abundance. Blue light reduced growth but maintained photosynthetic efficiency, whereas red light induced broad transcriptional changes. Untargeted metabolomics yielded 949 putatively annotated metabolites, including a feature tentatively assigned as phloroglucinol that did not differ significantly among treatments. Seven putative PKS-related transcripts, including four candidate type III PKS transcripts, and 26 putative ACC-related transcripts showed treatment-dependent expression patterns.

These findings identify candidate molecular and metabolic responses associated with altered light regimes but do not establish the biosynthetic regulation, downstream polymerization, or production optimization of structurally defined phlorotannins. The study was limited by the use of a non-specific Folin–Ciocalteu assay, MSI Level 2 metabolite annotations without authenticated-standard confirmation, single-time-point multi-omics sampling, and correlation-based integration. Targeted phlorotannin profiling, functional validation of candidate transcripts, enzyme activity measurements, and metabolic flux analyses will be necessary to determine whether the observed responses directly affect phlorotannin biosynthesis.

## Data Availability

The raw data supporting the conclusions of this article will be made available by the authors upon request.

## Supplementary Materials

The following supporting information can be downloaded at https://www.mdpi.com/article/10.3390/md24090330/s1, Figure S1: Volcano plots of differentially expressed genes (DEGs) under different light treatments; Figure S2: GO enrichment analysis of DEGs under different light regimes; Figure S3: Principal component analysis (PCA) of metabolites under different light treatments; Figure S4: Volcano plots of differential metabolites under different light treatments; Figure S5: Distribution of differential metabolites among light treatments; Figure S6: KEGG pathway enrichment analysis of differential metabolites under different light treatments; Figure S7: Hierarchical clustering heatmap of top 50 differential metabolites under different light treatments; Figure S8: Correlation analysis between KEGG pathway-annotated genes and metabolites under different light conditions.
