# Supplementary material for: Effects of Photoperiod and Light Quality on Phlorotannin Accumulation and Multi-Omics Responses in Sargassum muticum
Source: Mar Drugs. 2026 Sep 21;24(9):330. doi: 10.3390/md24090330 (PMC13608119; doi:10.3390/md24090330)
Supplement: Supplementary file 1 [file marinedrugs-24-00330-s001.zip › marinedrugs-4485340-supplementary.pdf]

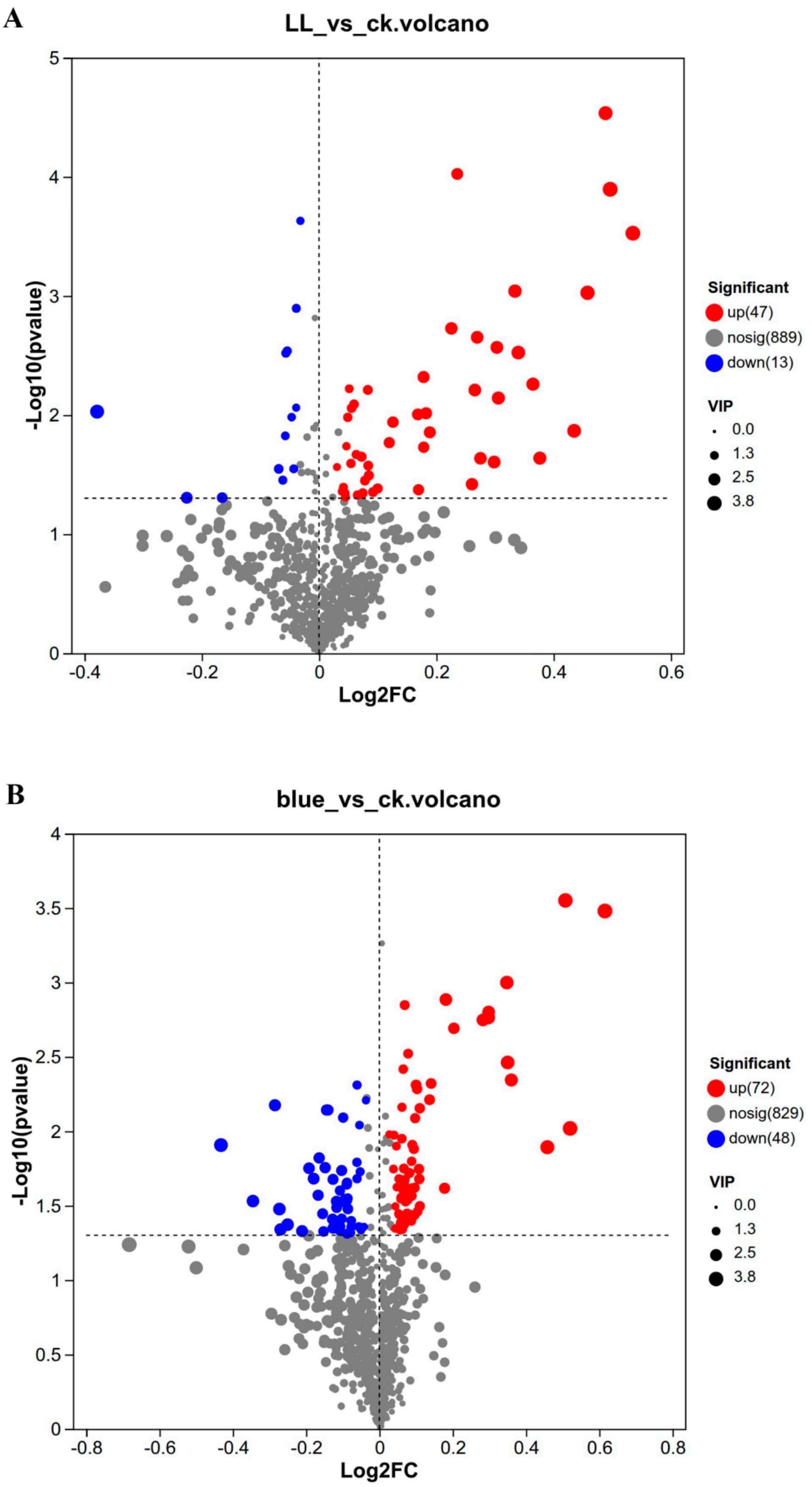
A
LL_vs_ck.volcano
-Log10(pvalue)
Log2FC
Significant
up(47)
nosig(889)
down(13)
VIP
0.0
1.3
2.5
3.8
B
blue_vs_ck.volcano
-Log10(pvalue)
Log2FC
Significant
up(72)
nosig(829)
down(48)
VIP
0.0
1.3
2.5
3.8

C

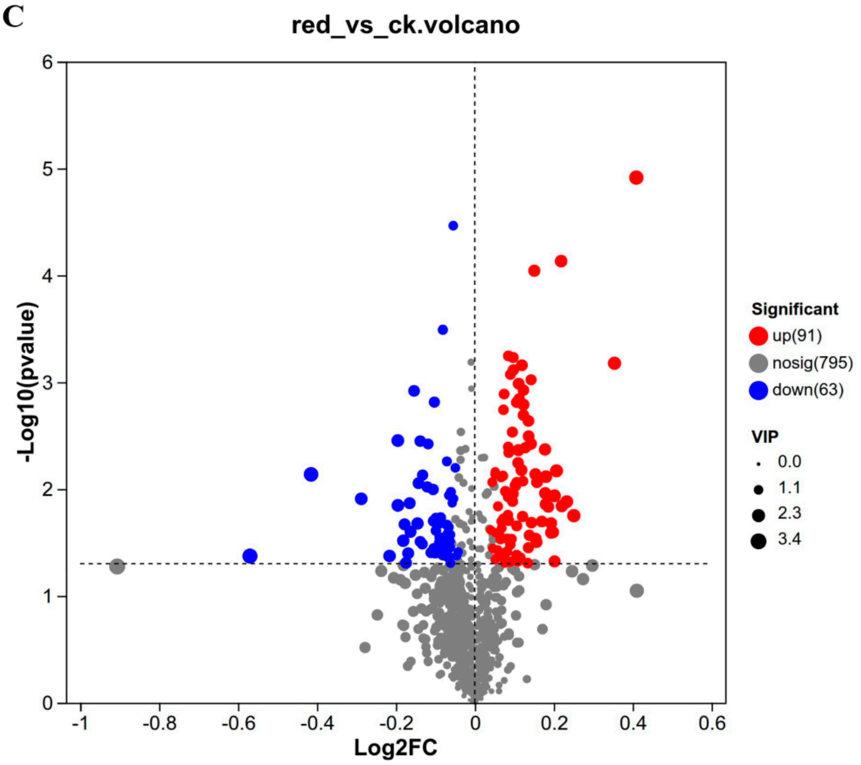

Figure S1. Volcano plots of differentially expressed genes (DEGs) under different light treatments. (**A**) Comparison between red light treatment group and control group (LL vs CK); (**B**) Comparison between blue light treatment group and control group (blue vs CK); (**C**) Comparison between long-day white light treatment group and control group (red vs CK). X-axis: log_2 fold change (Log2FC); Y-axis: -log_{10}(P-value). Red dots: up-regulated genes; blue dots: down-regulated genes; gray dots: non-significant genes. Dot size represents VIP scores from OPLS-DA analysis. Dashed lines indicate significance thresholds (|Log2FC| > 0, $P < 0.05$).

A

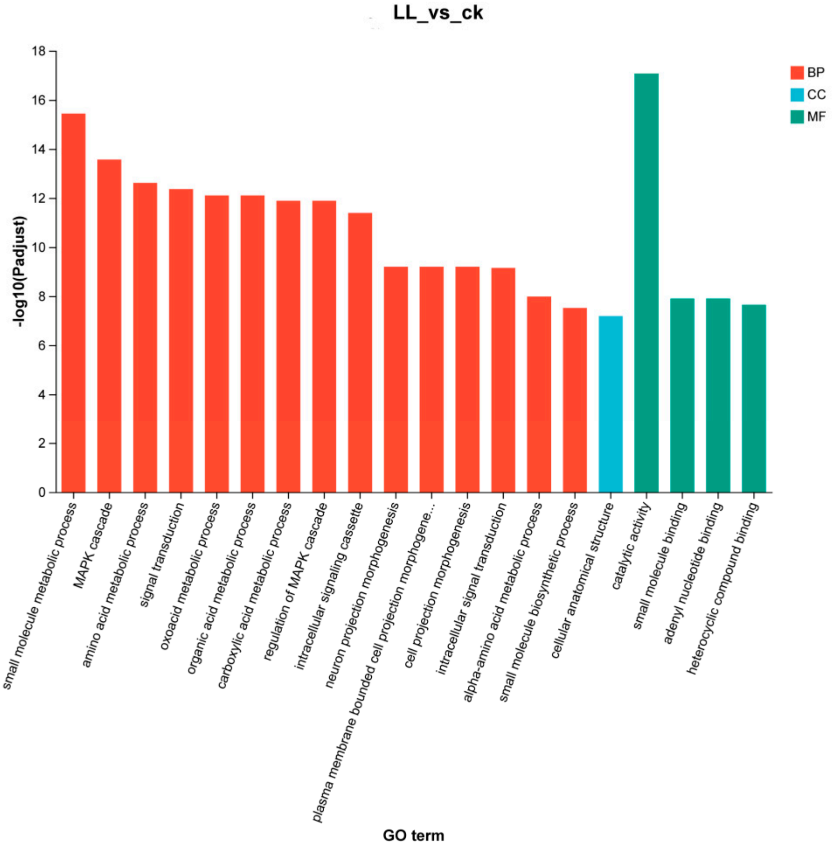
LL_vs_ck
-log10(Padjust)
0
2
4
6
8
10
12
14
16
18
BP
CC
MF
small molecule metabolic process
MAPK cascade
amino acid metabolic process
signal transduction
oxoacid metabolic process
organic acid metabolic process
carboxylic acid metabolic process
regulation of MAPK cascade
intracellular signaling cassette
neuron projection morphogenesis
plasma membrane bounded cell projection morphogene...
cell projection morphogenesis
intracellular signal transduction
alpha-amino acid metabolic process
small molecule biosynthetic process
cellular anatomical structure
catalytic activity
small molecule binding
adenyl nucleotide binding
heterocyclic compound binding
GO term

B

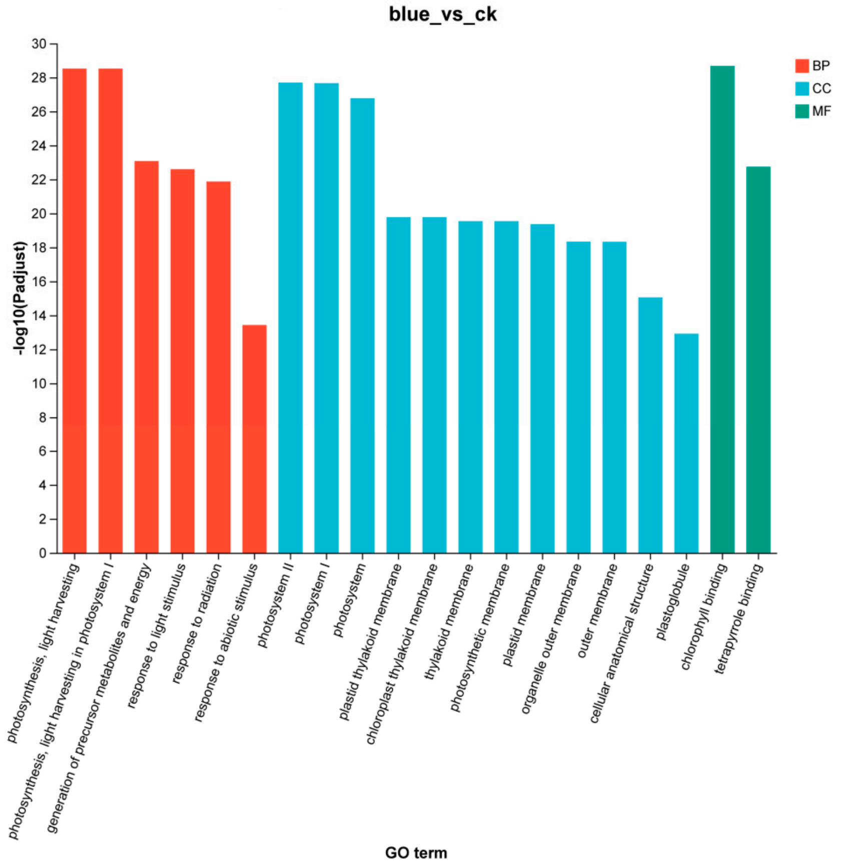
blue_vs_ck
-log10(Padjust)
0
2
4
6
8
10
12
14
16
18
20
22
24
26
28
30
BP
CC
MF
photosynthesis, light harvesting
photosynthesis, light harvesting in photosystem I
generation of precursor metabolites and energy
response to light stimulus
response to radiation
response to abiotic stimulus
photosystem II
photosystem I
photosystem
plastid thylakoid membrane
chloroplast thylakoid membrane
thylakoid membrane
photosynthetic membrane
plastid membrane
organelle outer membrane
outer membrane
cellular anatomical structure
plastoglobule
chlorophyll binding
tetrapyrrole binding
GO term

C

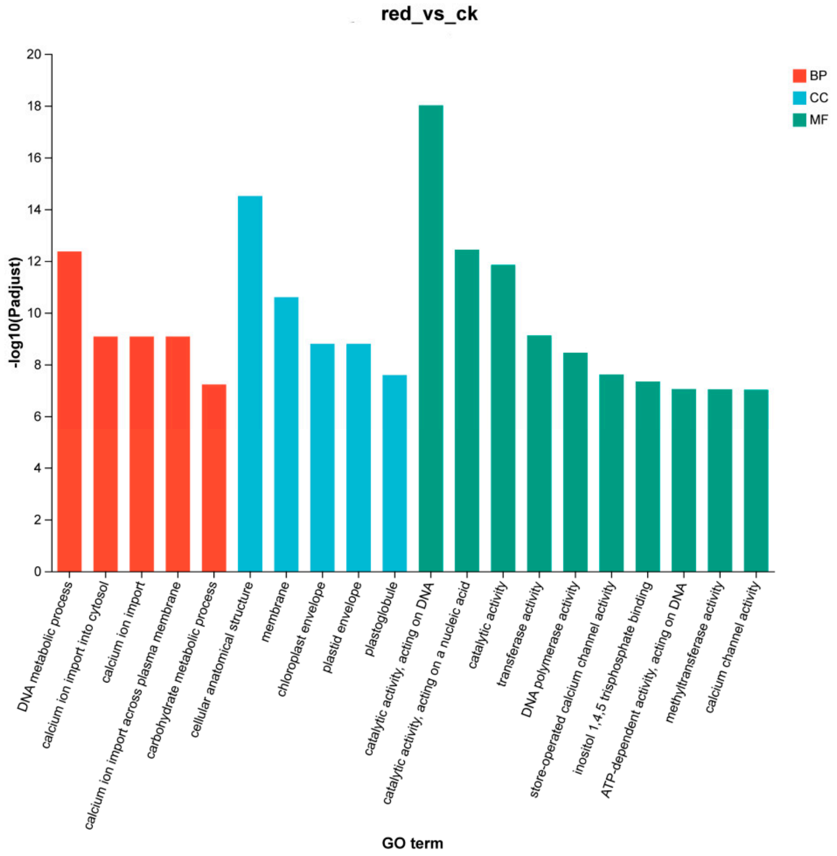

Figure S2. GO enrichment analysis of DEGs under different light regimes. The top 20 significantly enriched Gene Ontology (GO) terms are shown. The y-axis represents GO term names, and the x-axis represents the enrichment significance level ($-\log_{10}$Padjust value). Bar height indicates the degree of enrichment significance; smaller FDR values correspond to larger $-\log_{10}$(Padjust) values, indicating more significant enrichment. Three colors represent the three major GO categories: red for Biological Process (BP), blue for Cellular Component (CC), and green for Molecular Function (MF).

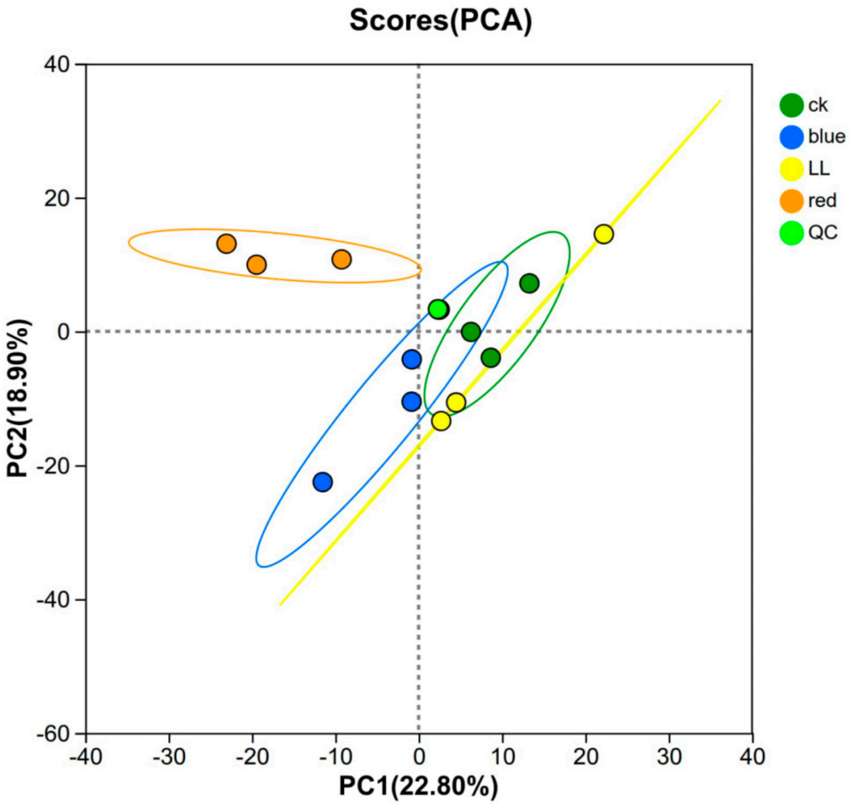

Figure S3. Principal component analysis (PCA) of metabolites under different light treatments. Principal component analysis was performed on metabolite data from *S. muticum* under different light quality treatments to evaluate metabolic profile differences among treatment groups. Each dot represents a biological replicate sample, and different colors represent different light quality treatment groups: blue for control group (white light, CK), red for red light treatment group (red), yellow for blue light treatment group (blue), and green for long-day white light treatment group (LL). The x-axis represents the first principal component (PC1), and the y-axis represents the second principal component (PC2).

A

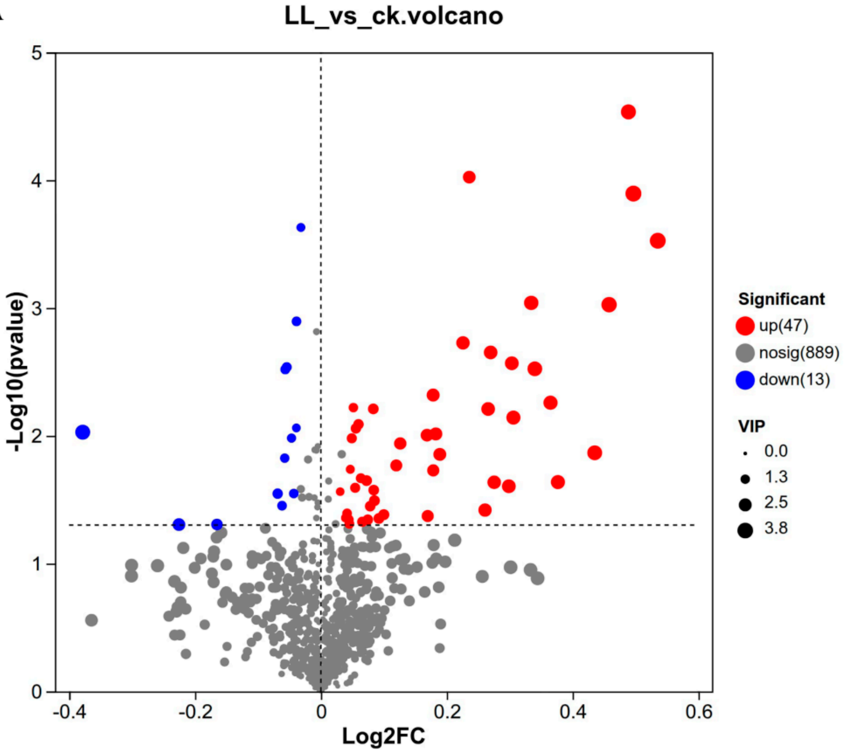
LL_vs_ck.volcano
-Log10(pvalue)
Log2FC
Significant
up(47)
nosig(889)
down(13)
VIP
0.0
1.3
2.5
3.8

B

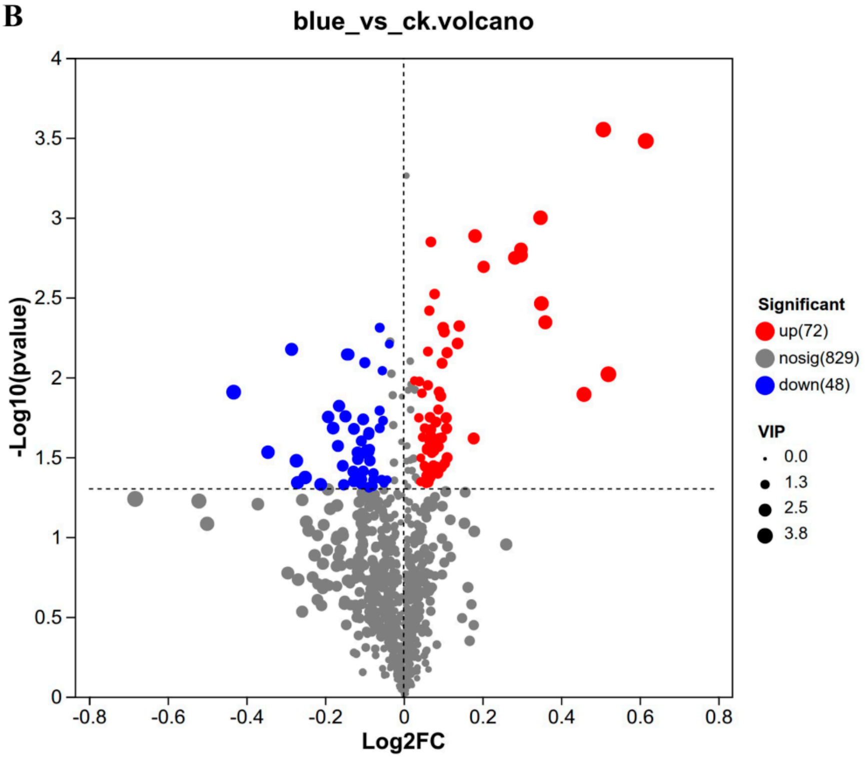
blue_vs_ck.volcano
-Log10(pvalue)
Log2FC
Significant
up(72)
nosig(829)
down(48)
VIP
0.0
1.3
2.5
3.8

C

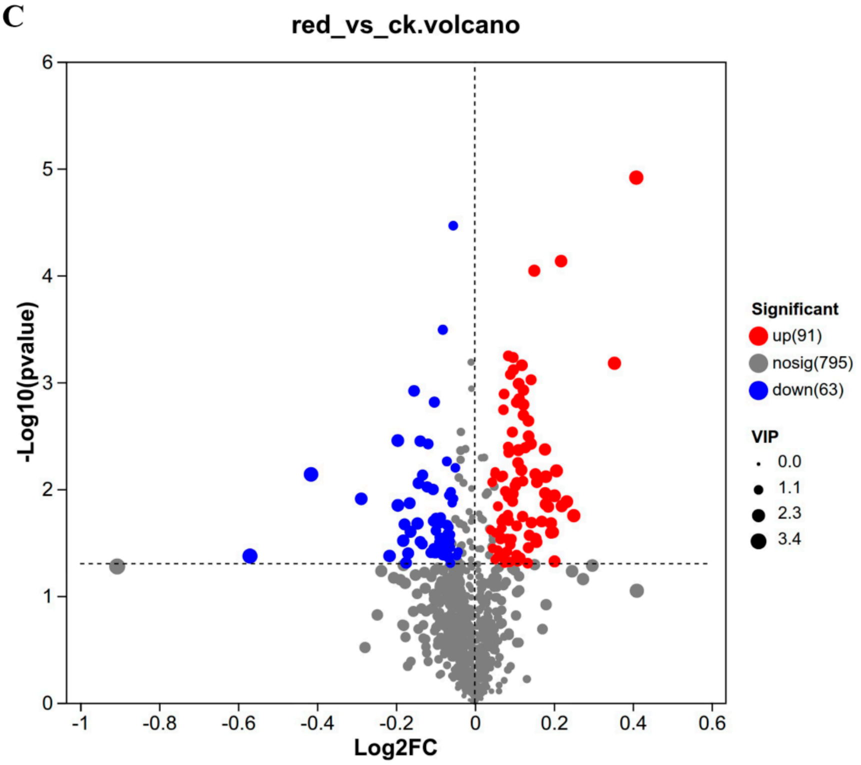

Figure S4. Volcano plots of differential metabolites under different light treatments. (A) Comparison between red light treatment group and control group (LL vs CK); (B) Comparison between blue light treatment group and control group (blue vs CK); (C) Comparison between long-day white light treatment group and control group (red vs CK). The x-axis represents the log2 fold change in metabolite expression levels (Log2FC), and the y-axis represents the negative logarithm of significance (-Log10(p-value)). Red dots indicate significantly up-regulated metabolites (up), blue dots indicate significantly down-regulated metabolites (down), and gray dots indicate metabolites with no significant differences (nosig). The size of the dots represents the VIP value (Variable Importance in Projection), with larger VIP values indicating greater importance of the metabolite in inter-group differences. Dashed lines indicate the screening thresholds (*P* value < 0.05, |Log2FC| > 0).

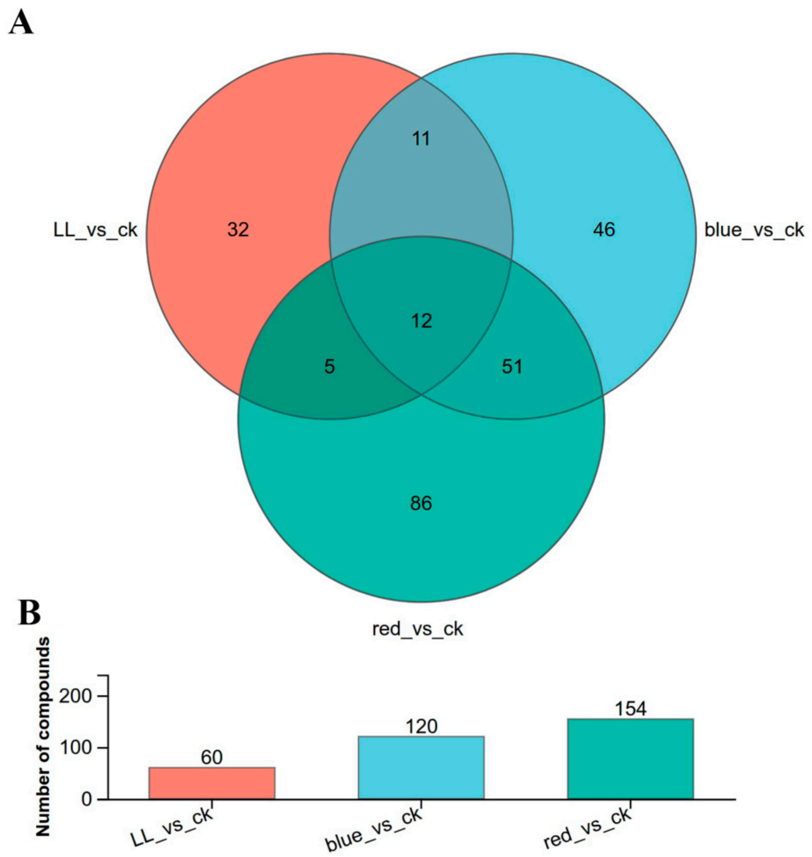

Figure S5. Distribution of differential metabolites among light treatments. (A) Venn diagram showing overlaps of differential metabolites. The red circle represents red vs CK, the blue circle represents blue vs CK, and the green circle represents LL vs CK. Numbers indicate unique or shared metabolites. (B) Bar chart displaying the total number of differential metabolites identified in each comparison.

A

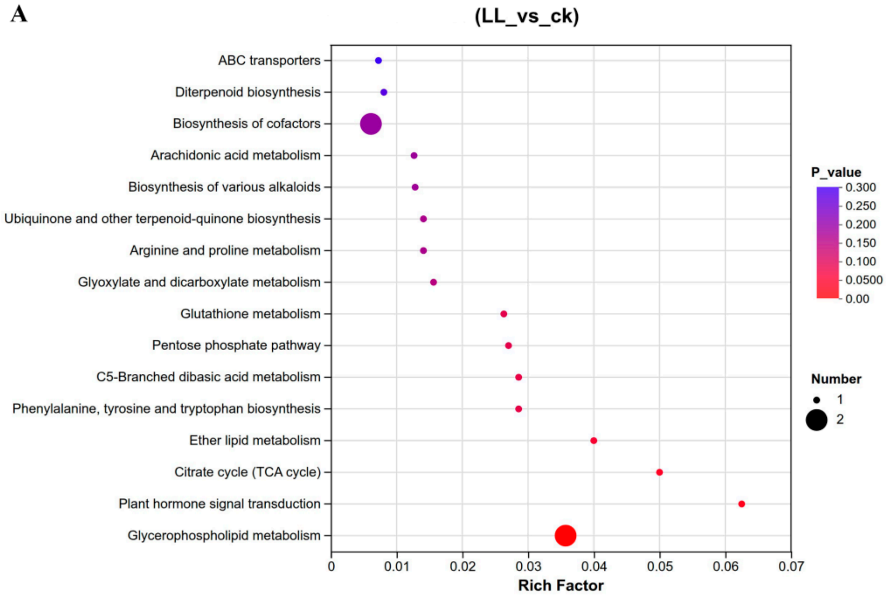
(LL_vs_ck)
ABC transporters
Diterpenoid biosynthesis
Biosynthesis of cofactors
Arachidonic acid metabolism
Biosynthesis of various alkaloids
Ubiquinone and other terpenoid-quinone biosynthesis
Arginine and proline metabolism
Glyoxylate and dicarboxylate metabolism
Glutathione metabolism
Pentose phosphate pathway
C5-Branched dibasic acid metabolism
Phenylalanine, tyrosine and tryptophan biosynthesis
Ether lipid metabolism
Citrate cycle (TCA cycle)
Plant hormone signal transduction
Glycerophospholipid metabolism
0
0.01
0.02
0.03
0.04
0.05
0.06
0.07
Rich Factor
P_value
0.300
0.250
0.200
0.150
0.100
0.0500
0.00
Number
1
2

B

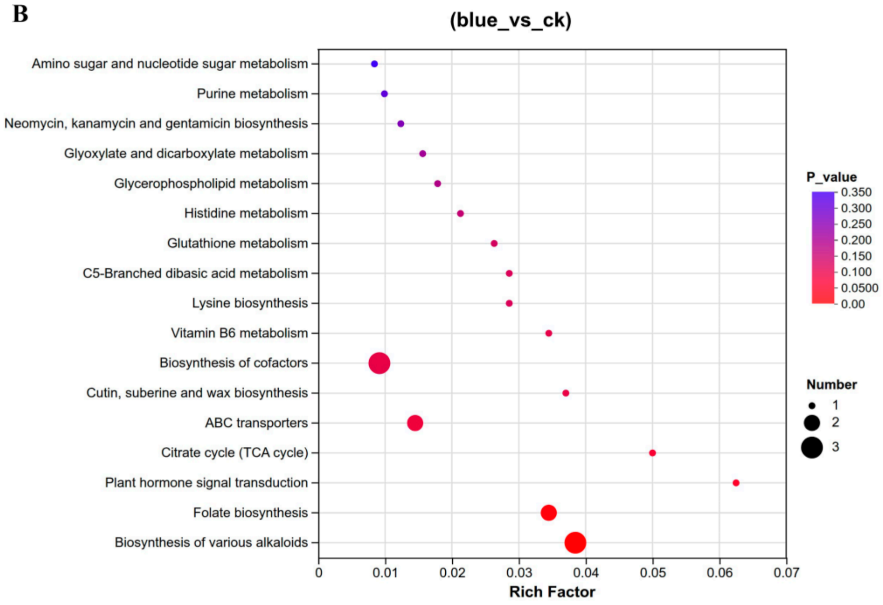
(blue_vs_ck)
Amino sugar and nucleotide sugar metabolism
Purine metabolism
Neomycin, kanamycin and gentamicin biosynthesis
Glyoxylate and dicarboxylate metabolism
Glycerophospholipid metabolism
Histidine metabolism
Glutathione metabolism
C5-Branched dibasic acid metabolism
Lysine biosynthesis
Vitamin B6 metabolism
Biosynthesis of cofactors
Cutin, suberine and wax biosynthesis
ABC transporters
Citrate cycle (TCA cycle)
Plant hormone signal transduction
Folate biosynthesis
Biosynthesis of various alkaloids
0
0.01
0.02
0.03
0.04
0.05
0.06
0.07
Rich Factor
P_value
0.350
0.300
0.250
0.200
0.150
0.100
0.0500
0.00
Number
1
2
3

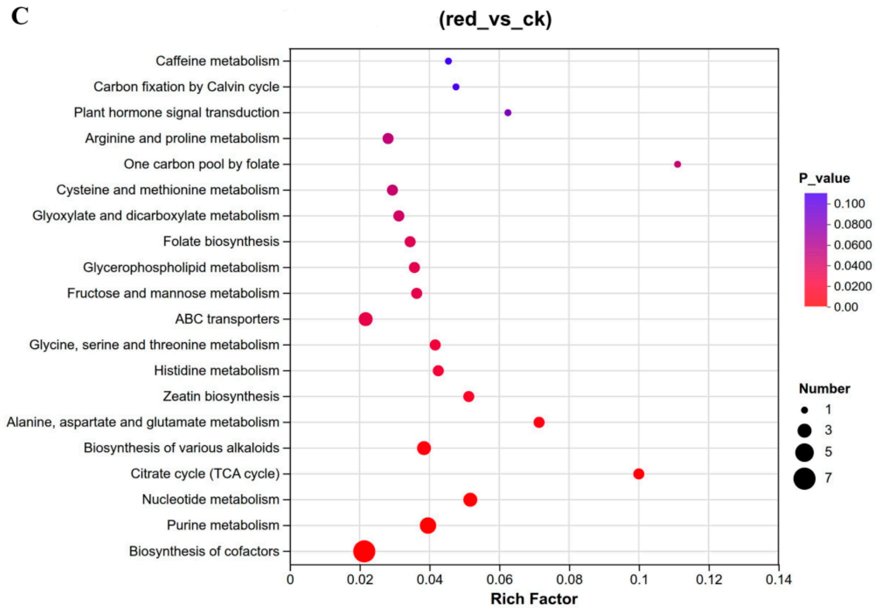

Figure S6. KEGG pathway enrichment analysis of differential metabolites under different light treatments. Bubble plots showing KEGG pathway enrichment for differential metabolites in three comparisons: (A) LL vs CK, (B) blue vs CK, and (C) red vs CK. Only pathways with corrected *P* < 0.05 are shown. The x-axis shows Rich Factor (ratio of differential metabolites in a pathway to total annotated metabolites in that pathway), with higher values indicating stronger enrichment. The y-axis lists KEGG pathways ranked by Rich Factor. Bubble size represents the number of differential metabolites (Number), and the bubble color indicates enrichment significance (P value).

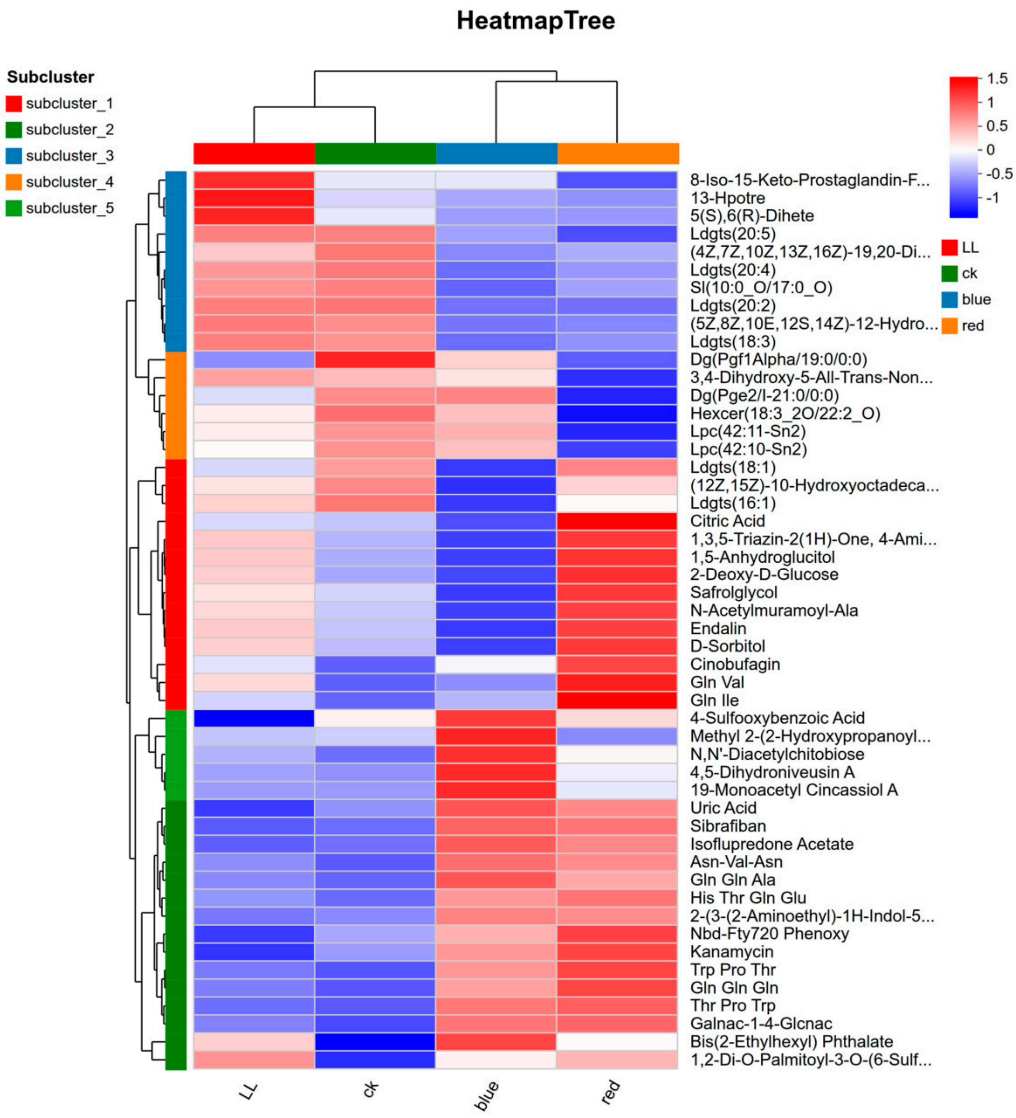

Figure S7. Hierarchical clustering heatmap of top 50 differential metabolites under different light treatments. Hierarchical clustering analysis was performed on the top 50 differential metabolites (ranked by VIP scores from PLS-DA analysis) to reveal treatment-specific expression patterns across 4 treatments. Each row represents an individual metabolite, and each column represents a sample. The color scale indicates standardized relative abundance values (Z-score normalization), with red representing high expression, blue representing low expression, and white representing intermediate expression levels. Sample groups are indicated by colored blocks at the top: CK (orange, white light control), Red (orange, red light treatment), Blue (blue, blue light treatment), and LL (red, long photoperiod treatment). Metabolite cluster groups (I–V) are indicated by colored blocks on the left side, representing distinct expression patterns.

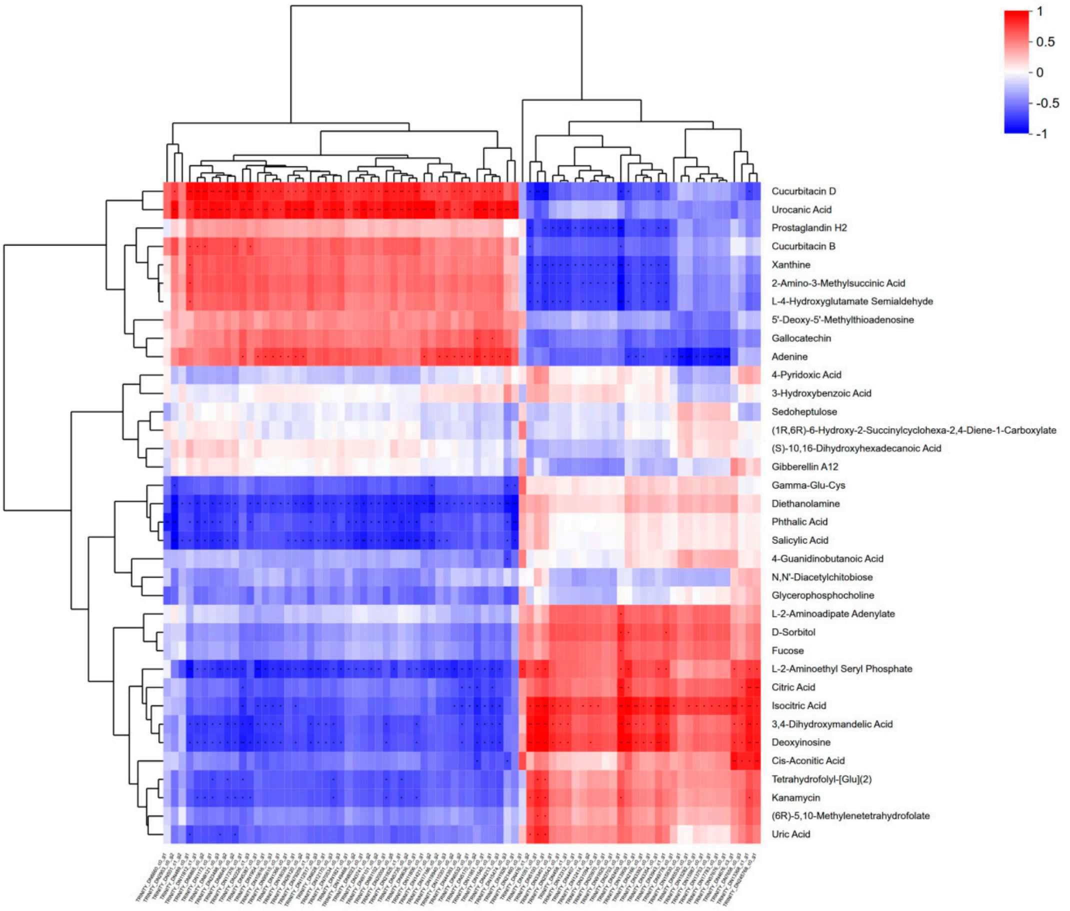

Figure S8. Correlation analysis between KEGG pathway-annotated genes and metabolites under different light conditions. Heatmap displaying Pearson correlation coefficients between between 79 KEGG-annotated DEGs (x-axis) and 36 KEGG-mapped metabolites (y-axis) across four light treatments (white light 12L:12D control, extended photoperiod 20L:4D, blue light, red light; n = 3 biological replicates per treatment). The color scale ranges from −1 (blue, negative correlation) to +1 (red, positive correlation). Hierarchical clustering was performed using Euclidean distance. Black dots indicate statistically significant correlations (*P* < 0.05).
